# NMMHC IIA triggers neuronal autophagic cell death by promoting F-actin-dependent ATG9A trafficking in cerebral ischemia/reperfusion

**DOI:** 10.1038/s41419-020-2639-1

**Published:** 2020-06-08

**Authors:** Guangyun Wang, Tiezheng Wang, Yang Hu, Jieman Wang, Yan Wang, Yuanyuan Zhang, Fang Li, Wentao Liu, Yang Sun, Boyang Yu, Junping Kou

**Affiliations:** 1 State Key Laboratory of Natural Products, Jiangsu Key Laboratory of TCM Evaluation and Translational Research, Department of Pharmacology of Chinese Material Medica, School of Traditional Chinese Pharmacy, China Pharmaceutical University, Nanjing, 211198 China; 2 Department of Neurology, University of California, Davis, School of Medicine and Shriners Hospital, Sacramento, CA 95817 Berkeley, USA; 3 Department of Pharmacology, Jiangsu Key Laboratory of Neurodegeneration, Nanjing Medical University, Nanjing, 210029 China; 4 State Key Laboratory of Pharmaceutical Biotechnology, Deparment of Biotechnology and Pharmaceutical Sciences, School of Life Sciences, Nanjing University, Nanjing, 210023 China; 5 State Key Laboratory of Natural Products, Jiangsu Key Laboratory of TCM Evaluation and Translational Research, Department of Resource and Developmemt of Chinese Material Medica, School of Traditional Chinese Pharmacy, China Pharmaceutical University, Nanjing, 211198 China

**Keywords:** Cellular neuroscience, Stroke

## Abstract

Previous findings have shown that non-muscle myosin heavy-chain IIA (NMMHC IIA) is involved in autophagy induction triggered by starvation in *D. melanogaster*; however, its functional contribution to neuronal autophagy remains unclear. The aim of this study is to explore the function of NMMHC IIA in cerebral ischemia-induced neuronal autophagy and the underlying mechanism related to autophagy-related gene 9A (ATG9A) trafficking. Functional assays and molecular mechanism studies were used to investigate the role of NMMHC IIA in cerebral ischemia-induced neuronal autophagy in vivo and in vitro. A middle cerebral artery occlusion (MCAO) model in mice was used to evaluate the therapeutic effect of blebbistatin, a myosin II ATPase inhibitor. Herein, either depletion or knockdown of NMMHC IIA led to increased cell viability in both primary cultured cortical neurons and pheochromocytoma (PC12) cells exposed to oxygen–glucose deprivation/reoxygenation (OGD/R). In addition, NMMHC IIA and autophagic marker LC3B were upregulated by OGD/R, and inhibition of NMMHC IIA significantly reduced OGD-induced neuronal autophagy. Furthermore, NMMHC IIA-induced autophagy is through its interactions with F-actin and ATG9A in response to OGD/R. The NMMHC IIA–actin interaction contributes to ATG9A trafficking and autophagosome formation. Inhibition of the NMMHC IIA–actin interaction using blebbistatin and the F-actin polymerization inhibitor cytochalasin D significantly suppressed ATG9A trafficking and autophagy induction. Furthermore, blebbistatin significantly improved neurological deficits and infarct volume after ischemic attack in mice, accompanied by ATG9A trafficking and autophagy inhibition. These findings demonstrate neuroprotective effects of NMMHC IIA inhibition on regulating ATG9A trafficking-dependent autophagy activation in the context of cerebral ischemia/reperfusion.

## Introduction

Ischemic stroke is one of the primary causes of disability and death worldwide^1^. Although many devastating cascades^2–4^ have been shown to be associated with ischemic stroke, the precise mechanism underlying ischemic neuronal injury has not been fully elucidated. Autophagy is an important evolutionarily conserved process in eukaryotes for the turnover of intracellular substances^5–7^. Accumulating evidence indicates that autophagy is indeed involved in the pathophysiological changes that occur in ischemic stroke^8,9^. Neurons are very sensitive to ischemic stimulation, and neuronal autophagy is considered to be a manner of cell death that causes harmful effects in cerebral ischemia. Knockdown of Atg7 or Beclin 1 using shRNAs reduces neuronal autophagy and protects against kainite plus hypoxia-induced excitotoxicity in rats^10^. In vivo experiments also showed that injection of 3-methyladenine (3-MA) into the lateral ventricle after cerebral ischemia significantly decreased both neuronal autophagy and the lesion volume^11^. Therefore, exploring relevant targets to inhibit neuronal autophagic cell death could potentially aid in the prevention or the treatment of ischemic stroke.

Recent studies have proven that the formation and transport of autophagosomes require several cytoskeletal components, including actin cytoskeleton and myosin motor proteins^12,13^. Actin cytoskeleton provides a filament network for the delivery of autophagic membrane components from different cellular compartments to the autophagosome. ATG9, an autophagy-related gene, is required for transportation of membranous components and autophagosome formation. Movement of ATG9 was tracked over time in living cells with a real-time fluorescence microscopy, and the results showed that Arp2 (an actin-related protein) briefly colocalizes with Atg9 and directly regulates the movement of ATG9^14^. In addition, a pool of Atg11 mediates the anterograde transport of ATG9 to the preautophagosomal structure (PAS) that is dependent on the actin cytoskeleton during yeast vegetative growth^15^. These results demonstrate that actin filaments are essential for autophagy and the movement of Atg9 between the peripheral sites and the PAS^16^. Moreover, actin filaments and myosin constitute the actomyosin system. It has been reported that one of the myosin isofmors, non-muscle myosin heavy-chain IIA (NMMHC IIA) is involved in the delivery of ATG9-riched membranes in the early stages of autophagy to help the initial formation of the autophagosome. During starvation, UNC51-like kinase 1 (ULK1/Atg1) promotes phosphorylation-dependent activation of NMMHC II to regulate ATG9A trafficking from the trans-Golgi network (TGN) to PAS, facilitating autophagosome formation^17^. However, the precise relationship among myosin II, actin, and ATG9 in autophagosome formation remains unclear.

NMMHC IIA is one of the three different myosin II isoforms (NMMHC IIA, IIB, and IIC) that is encoded by myosin heavy 9 (MYH9)^18^. NMMHC IIA is abundantly expressed and accomplishes many functions, including adhesion, cell migration, and translocation^19,20^. Increasing studies have confirmed its important role in human health and disease, including our previous work on its critical regulation of venous thrombosis^21,22^. In the brain, it has been reported clinically that one patient with MYH9 alteration experienced ischemic stroke^23^. NMMHC IIA also has been identified to participate in blood–brain barrier dysfunction in ischemia stroke^24^, and the NMMHC IIA–actin interaction mediates oxidative stress-induced neuronal apoptosis^25^. In summary, NMMHC IIA might represent a potential target in ischemic stroke, but its role in ischemia-induced neuronal autophagic cell death and the underlying mechanism are essentially uncharacterized.

Herein, we demonstrate that NMMHC IIA induces neuronal autophagic cell death by interacting with F-actin and ATG9A. The NMMHC IIA–actin interaction promotes ATG9A trafficking and autophagy formation in cerebral ischemia/reperfusion. This study provides some new insights into the mechanisms of cerebral ischemia/reperfusion-induced neuronal autophagic cell death and paves the way for the development of improved treatments for ischemia stroke.

## Results

### NMMHC IIA inhibition prevents OGD/R-induced neuronal injury

**Fig. 1:**
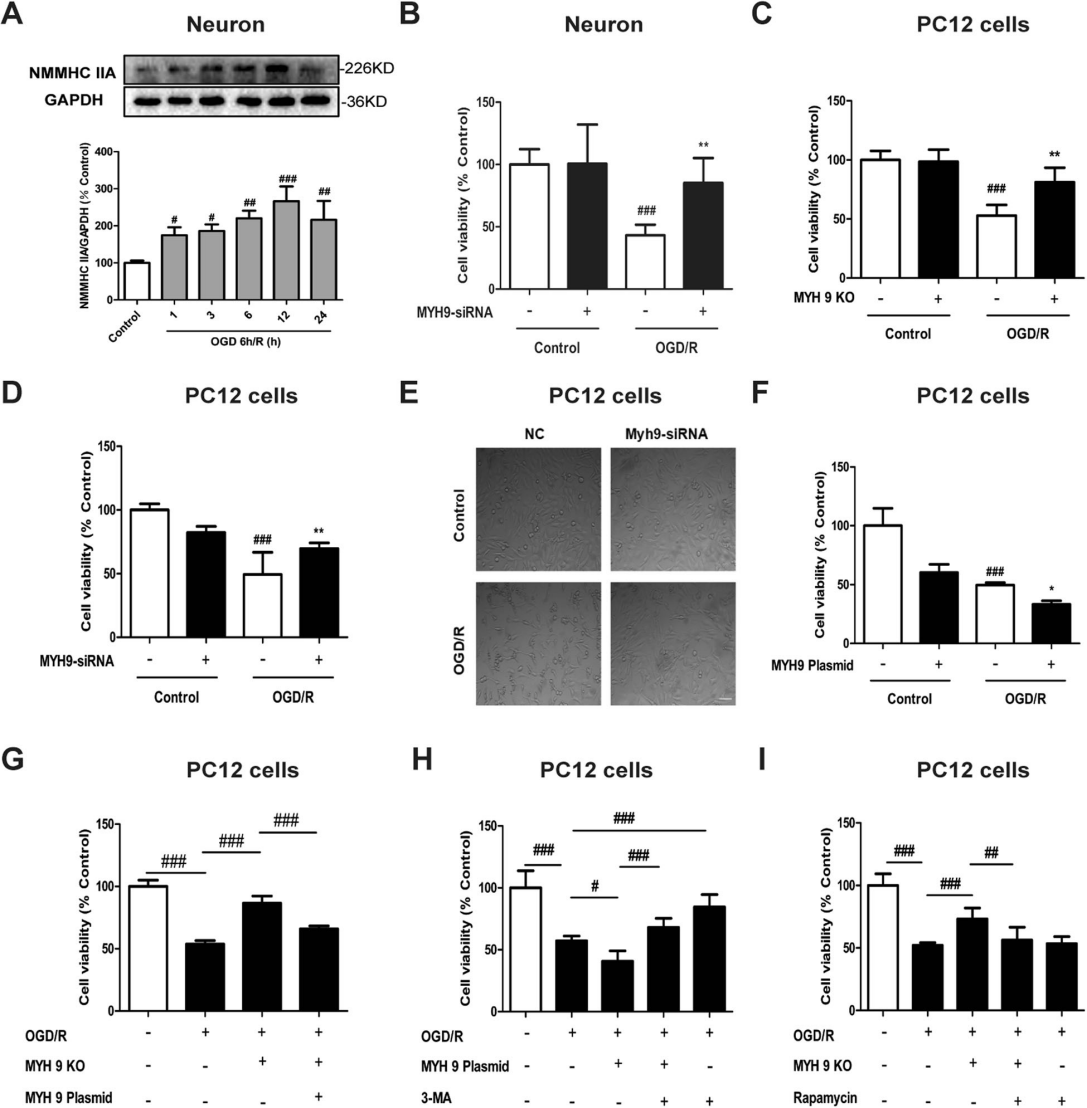
NMMHC IIA inhibition prevents OGD/R-induced neuronal injury. **a** Primary cortical neurons were treated with 6 h of OGD, and expression of NMMHC IIA at different time points of reoxygenation was determined by western blot. **b** Primary cortical neurons were transfected with siRNAs against NMMHC IIA, and cell viability was evaluated by MTT after OGD/R treatment. **c** The myh9 gene was deleted using the CRISPR/Cas9 system in PC12 cells. After OGD/R treatment, cell viability was evaluated by MTT assay. KO knockout. **d**, **e** PC12 cells were transfected with siRNAs against NMMHC IIA, and cell viability and cell morphology were evaluated after OGD/R treatment. NC negative control. **f** PC12 cells were transfected with MYH9 plasmid for 48 h, and cell viability was depicted followed by OGD/R treatment. All data are presented as the means ± SD, *n* = 6. ^###^*P* < 0.001 vs. control group; ^*^P < 0.05 and ^**^P < 0.01 vs. OGD/R-treated group. **g** The MYH9 gene was deleted using the CRISPR/Cas9 system, and then PC12 cells were transfected with the MYH9 plasmid. Cell viability was detected by MTT assay after transfection. **h** Treatment with an autophagy inhibitor, 3-MA (3 mM), during OGD/R in MYH9-overexpressing cells. Cell viability was detected by MTT. **i** Treatment with an autophagy inducer, rapamycin (0.1 μM), during OGD/R in myh9 knockout cells, and then cell viability was detected by MTT. PC12 cells and neurons were treated with 6 h of OGD and then reoxygenated for 6 h. All data are presented as the means ± SD, *n* = 6. ^#^*P* < 0.05, ^##^*P* < 0.01 and ^###^*P* < 0.001.

As shown in Supplementary Fig. S1A, we assessed the neuronal marker MAP2 by immunofluorescence. Consistent with previous reports, most of the cells expressed the neuronal marker MAP2, and MAP2 fluorescence decreased in neurons under OGD/R induction. Western blot result revealed that OGD/R significantly increased levels of NMMHC IIA in neurons (Fig. 1a). However, there were no obvious changes in NMMHC IIB or IIC (Supplementary Fig. S3A, B). Similar results were also found in PC12 cells (Supplementary Fig. S3D–F). It has been suggested that NMMHC IIA may play an important role in OGD/R-induced neuronal damage. NMMHC IIA knockdown in cortical neurons has no effect on cell viability. However, under OGD/R condition, NMMHC IIA knockdown increased cell viability compared with negative control cells (Fig. 1b; Supplementary Fig. S1B). To further confirm the effects of NMMHC IIA, the CRISPR/Cas9 system was used to knockout the MYH9 gene in PC12 cells, and NMMHC IIA was completely depleted compared with wild-type (WT) cells by western blot analysis (Supplementary Fig. S1C). Consistently, knockout or knockdown of NMMHC IIA in PC12 cells significantly increased cell viability after OGD/R (Fig. 1c, d; Supplementary Fig. S1D). NMMHC IIA knockdown also protected PC12 cells from OGD/R-induced cell contraction and membrane blebbing (Fig. 1e). On the contrary, NMMHC IIA overexpression aggravated OGD/R-induced cell damage compared with negative control (Fig. 1f; Supplementary Fig. S1E). Next, we transfected MYH9 overexpression plasmid into MYH9 knockout PC12 cells to rescue CRISPR/Cas9 knockout MYH9 activity. MYH9 overexpression plasmid transfection remarkably increased the expression of NMMHC IIA in MYH9 knockout PC12 cells (Supplementary Fig. S1F), and substantially reversed the protective effects of NMMHC IIA knockout on OGD/R-induced cell injury (Fig. 1g). These results demonstrate NMMHC IIA contributes to OGD/R-induced neuronal damage.

### NMMHC IIA inhibition attenuates OGD/R-induced autophagy in neurons

To confirm autophagy as a mechanism for OGD/R-induced cell death, the effects of critical proteins (Beclin 1 and Atg7) for autophagy were examined on OGD/R-induced cell death. RNAi knockdown reduced protein levels of Beclin 1 and Atg7 by ~50% (Supplementary Fig. S2A, B), significantly attenuating OGD/R-induced cell damage (Supplementary Fig. S2C).

To study whether autophagy is associated with NMMHC IIA-induced neuronal damage, PC12 cells transfected with NMMHC IIA overexpression plasmid were treated or not treated with autophagy inhibitor, 3-MA. As shown in Fig. 1h, 3-MA significantly reduced cell death induced by OGD/R, indicating autophagy followed by autophagic cell death as a mechanism for OGD/R-induced cell death. Moreover, decreased cell viability induced by NMMHC IIA overexpression was increased by 3-MA. In contrast, increased cell viability induced by NMMHC IIA knockout was significantly attenuated by rapamycin treatment (Fig. 1i). These results suggest that NMMHC IIA inhibition prevents OGD/R-induced neuronal autophagic cell death.

**Fig. 2:**
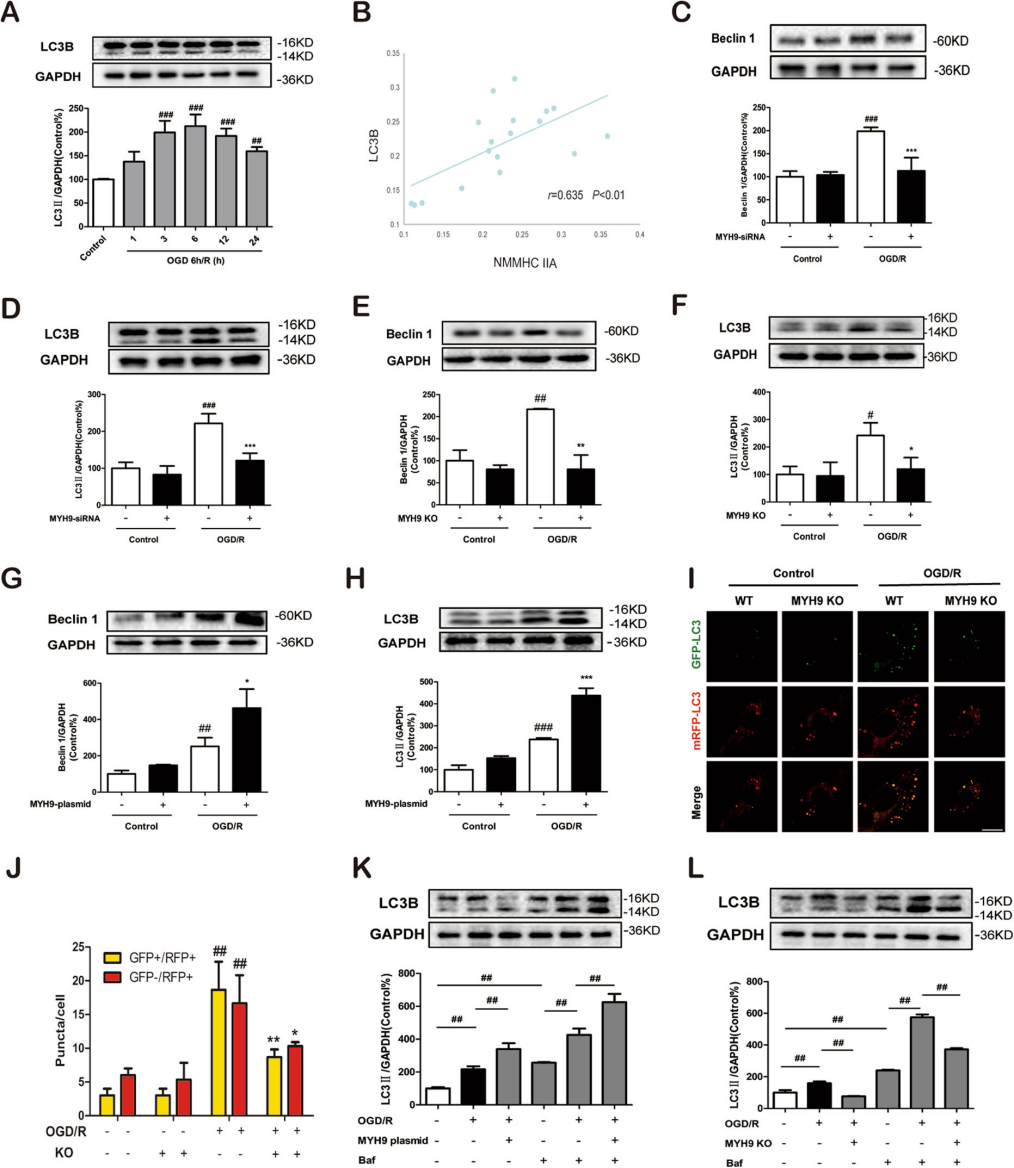
NMMHC IIA inhibition attenuates autophagy in PC12 cells and primary cortical neurons in response to OGD/R. **a** Primary cortical neurons were exposed to OGD/R, and expression of LC3B at different time points of reoxygenation was determined by western blot. **b** Correlation analysis of NMMHC IIA expression with LC3B in neurons in response to OGD/R. **c**, **d** Primary cortical neurons were transfected with siRNAs against NMMHC IIA, and the expression of Beclin 1 and LC3B was determined by western blotting. **e**, **f** The MYH9 gene was deleted using the CRISPR/Cas9 system in PC12 cells. After OGD/R treatment, western blot analysis was used to detect the expression of Beclin 1 and LC3B-II. **g**, **h** PC12 cells were transfected with the MYH9 plasmid for 48 h, and Beclin and LC3B-II expression were assessed followed by OGD/R treatment. All data are presented as the means ± SD of three independent experiments. ^#^*P* < 0.05, ^##^*P* < 0.01, and ^###^*P* < 0.001 vs. the control group; ^*^P < 0.05, ^**^P < 0.01, and ^***^P < 0.001 vs. OGD/R-treated group. **i**, **j** PC12 cells were infected with an mRFP-GFP-LC3B adenovirus, and were then subjected to OGD/R. Bar: 10 µm. WT wild-type. **k**, **l** PC12 cells were treated with Baf after NMMHC IIA was overexpressed or deleted, and cell lysates were prepared for analyzing the LC3 expression. PC12 cells and neurons were treated with 6 h of OGD and then reoxygenated for 6 h. ^#^*P* < 0.05 and ^##^*P* < 0.01.

Next, we test whether NMMHC IIA regulates neuronal autophagy in our in vitro model. Western blot result revealed that exposure to OGD/R also upregulated levels of autophagy markers, including Beclin 1 and LC3B-II in neurons and PC12 cells (Fig. 2a; Supplementary Fig. S3C, G, H). Correlation analysis showed that NMMHC IIA expression was positively correlated with the autophagy-related protein LC3B in neurons exposed to OGD/R (*r* = 0.635, *P* < 0.001, Fig. 2b). To confirm the correlation of NMMHC IIA and autophagy, we knocked down MYH9 gene in neurons using siRNA. Knockdown of NMMHC IIA remarkably reduced expressions of LC3B and Beclin 1 in primary neurons exposed to OGD/R (Fig. 2c, d). Similarly, NMMHC IIA knockdown and knockout in PC12 cells also inhibited OGD/R-induced autophagy (Supplementary Fig. S4; Fig. 2e, f). Moreover, overexpression of NMMHC IIA in PC12 cells increased OGD/R-induced autophagy (Fig. 2g, h). After OGD/R exposure, Beclin 1 and LC3B-II were significantly increased in MYH9 re-overexpressed cells, compared with MYH9 KO cells (Supplementary Fig. S5). Taken together, these findings demonstrate that NMMHC IIA plays an important role in OGD/R-induced autophagy in neurons.

Indeed, the most accurate way to measure autophagy activity is with an autophagic flux assay defined as the new formation of autophagosomes and their subsequent fusion with the lysosome. To determine whether NMMHC IIA could promote autophagic flux in PC12 cells, mRFP-GFP-LC3 adenoviral vectors were used to evaluate the autophagic level treated with NMMHC IIA knockout. As shown in Figs. 2i, j, NMMHC IIA knockout reduced yellow puncta (autophagosome) and red puncta (autolysosome) in PC12 cells compared with WT cells under OGD/R condition, which demonstrated that NMMHC IIA inhibition was important for blocking autophagic flux. Moreover, NMMHC IIA overexpression resulted in increased LC3B levels in PC12 cells following Bafilomycin A1 (Baf) treatment compared with the model group treated with Baf (Fig. 2k). The opposing result was found in NMMHC IIA knockout PC12 cells (Fig. 2l), suggesting that NMMHC IIA is involved in early stage of autophagosome formation.

### NMMHC IIA interacts with F-actin and ATG9A during OGD/R in PC12 cells

**Fig. 3:**
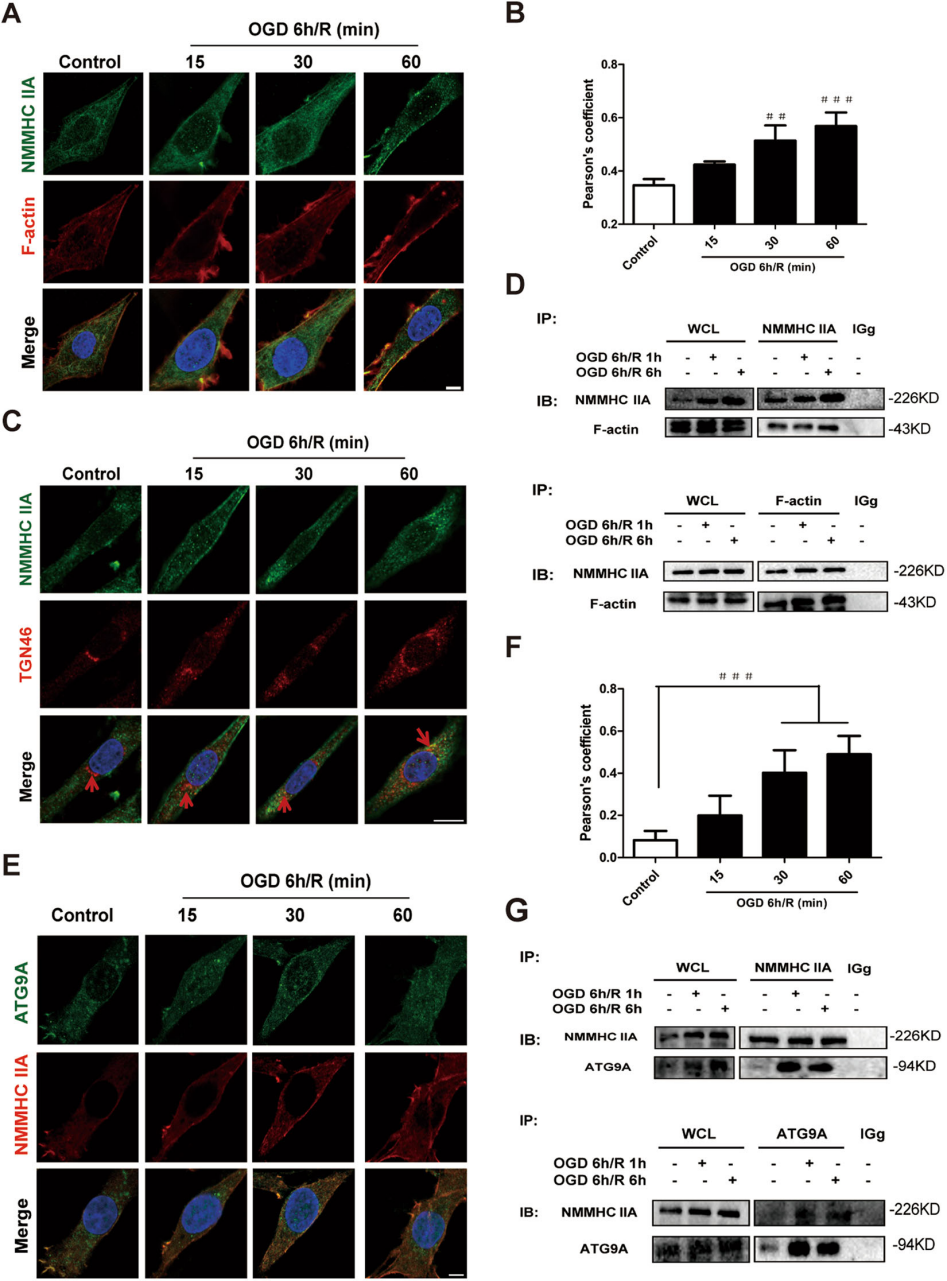
NMMHC IIA interacts with F-actin and ATG9A during OGD/R in PC12 cells. **a** PC12 cells treated with OGD/R were stained with NMMHC IIA (green), F-actin (red), and DAPI (blue). Bar: 5 µm. **b** Colocalization of NMMHC IIA with F-actin was evaluated by Pearson’s coefficients. **c** PC12 cells treated with OGD/R were stained with NMMHC IIA (green), TGN46 (red), and DAPI (blue). Bar: 5 µm. **d** Co-IP was used to detect protein interactions between actin and NMMHC IIA. WCL, whole-cell lysates. **e**, **f** PC12 cells treated with OGD/R were stained with Atg9A (green), NMMHC IIA (red), and DAPI (blue). Bar: 5 µm. Colocalization of NMMHC IIA with ATG9A was evaluated by Pearson’s coefficients. The results are expressed as the mean ± SD, *n* = 3. ^###^*P* < 0.001. **g** Co-IP was used to detect protein interactions between ATG9A and NMMHC IIA.

It has been reported that NMMHC IIA and F-actin interactiouring OGD/R in PC12 cells is responsible for oxidative stress-induced neuronal apoptosis^25^. Here, we investigate the mechanism of NMMHC IIA in regulating OGD/R-induced neuronal autophagy. Co-immunoprecipitation (Co-IP) and immunofluorescence analysis showed that NMMHC IIA and F-actin interaction increased in response to OGD/R (Fig. 3a–d). Pearson correlation coefficient illustrated that NMMHC IIA and F-actin were statistically significantly colocalized in response to OGD/R condition (Fig. 3b). In addition, NMMHC IIA was enriched in TGN in response to OGD/R conditions (Fig. 3c), which indicate that NMMHC IIA might play an important role in membrane trafficking from TGN. ATG9A is an autophagy-related gene that is required for the transportation of membranous components and autophagosomal formation. The interaction between NMMHC IIA and ATG9A was also increased in OGD/R condition (Fig. 3e–g). Similar results were obtained in the Duolink Proximity ligation assay (Supplementary Fig. S6). These findings demonstrate that NMMHC IIA interacts with F-actin and ATG9A during OGD/R in PC12 Cells.

### NMMHC IIA interacts with F-actin and ATG9A via its head and tail domains after OGD/R

**Fig. 4:**
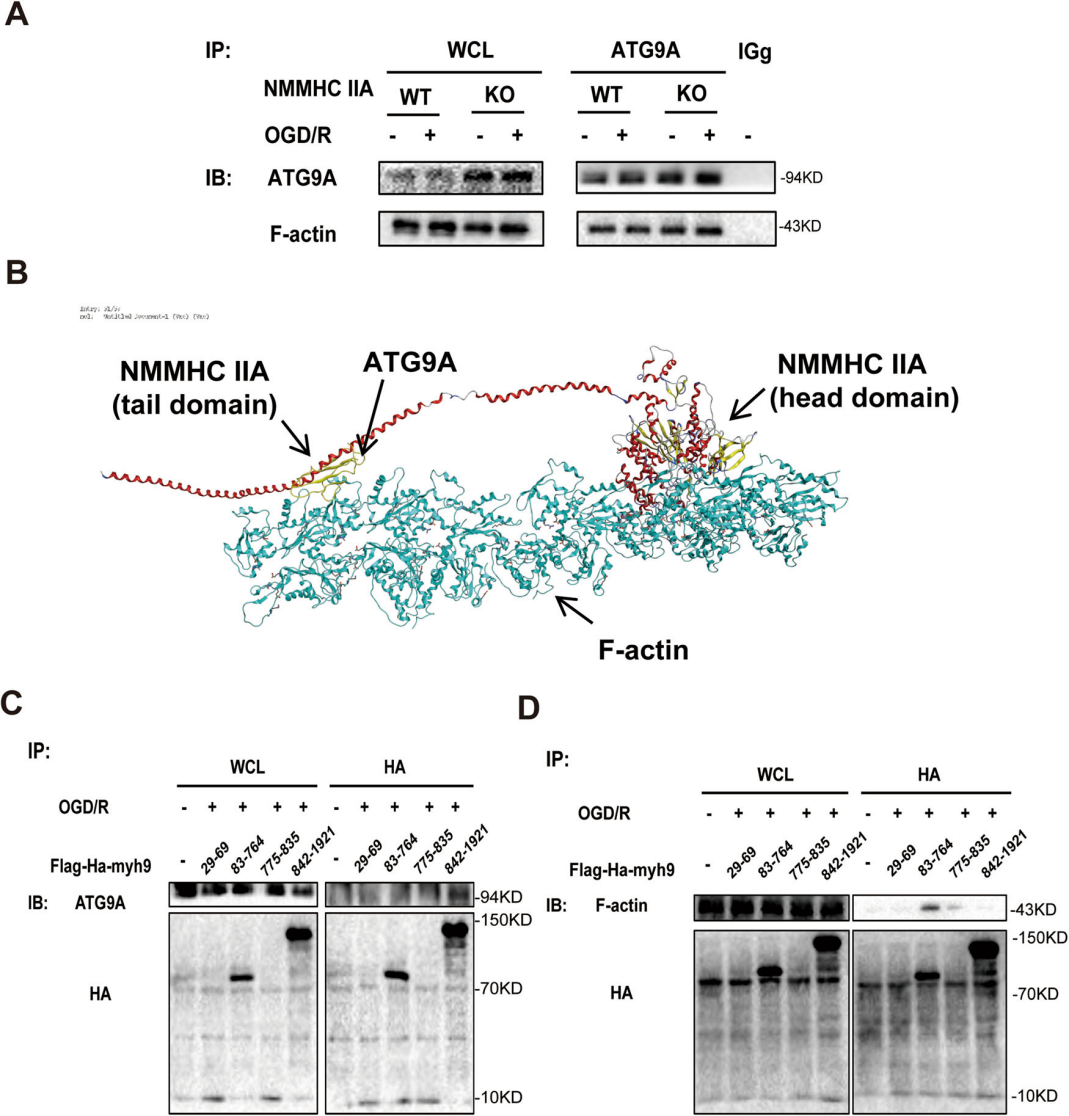
NMMHC IIA interacts with F-actin and ATG9A via its head and tail domains after OGD/R. **a** PC12 cells were treated with 6 h of OGD and then reoxygenated for 6 h. Co-IP was used to detect protein interactions between ATG9A and F-actin in NMMHC IIA knockout PC12 cells. **b** Schematic representations of NMMHC IIA interaction with F-actin and ATG9A. **c** Co-IP was used to detect protein interactions between ATG9A and different regions of NMMHC IIA in HEK293T cells. **d** Co-IP was used to detect protein interactions between F-actin and different regions of NMMHC IIA in HEK293T cells.

Our results also indicated that three proteins (NMMHC IIA, F-actin, and ATG9A) combine with one another by CO-IP in NMMHC IIA knockout PC12 cells (Fig. 4a). Molecular docking was used to predict the binding modes of NMMHC IIA, F-actin, and ATG9A. As shown in Fig. 4b, the tail domain of NMMHC IIA binds to ATG9A, and the head domain of NMMHC IIA interacts with F-actin. Next, to identify specific regions of NMMHC IIA required for interaction with ATG9A and F-actin, a series of HA-tagged NMMHC IIA fragment plasmids were transfected in HEK293T cells. As shown in Fig. 4c, d, the tail (aa. 842–1921) and head (aa. 83–764) domains of NMMHC IIA were essential for its interaction with ATG9A and F-actin, respectively. In view of the results above, we proposed a possible mechanism for NMMHC IIA-mediated autophagy induced by OGD/R. Upon OGD/R, the tail domain of NMMHC IIA binds to ATG9A, and at the same time the head domain of NMMHC IIA interacts with F-actin to provide power for ATG9A trafficking from the TGN and forming autophagosomes (Fig. 4b).

### Inhibition of the NMMHC IIA–actin interaction alleviates ATG9A trafficking and neuronal autophagic cell death during OGD/R

**Fig. 5:**
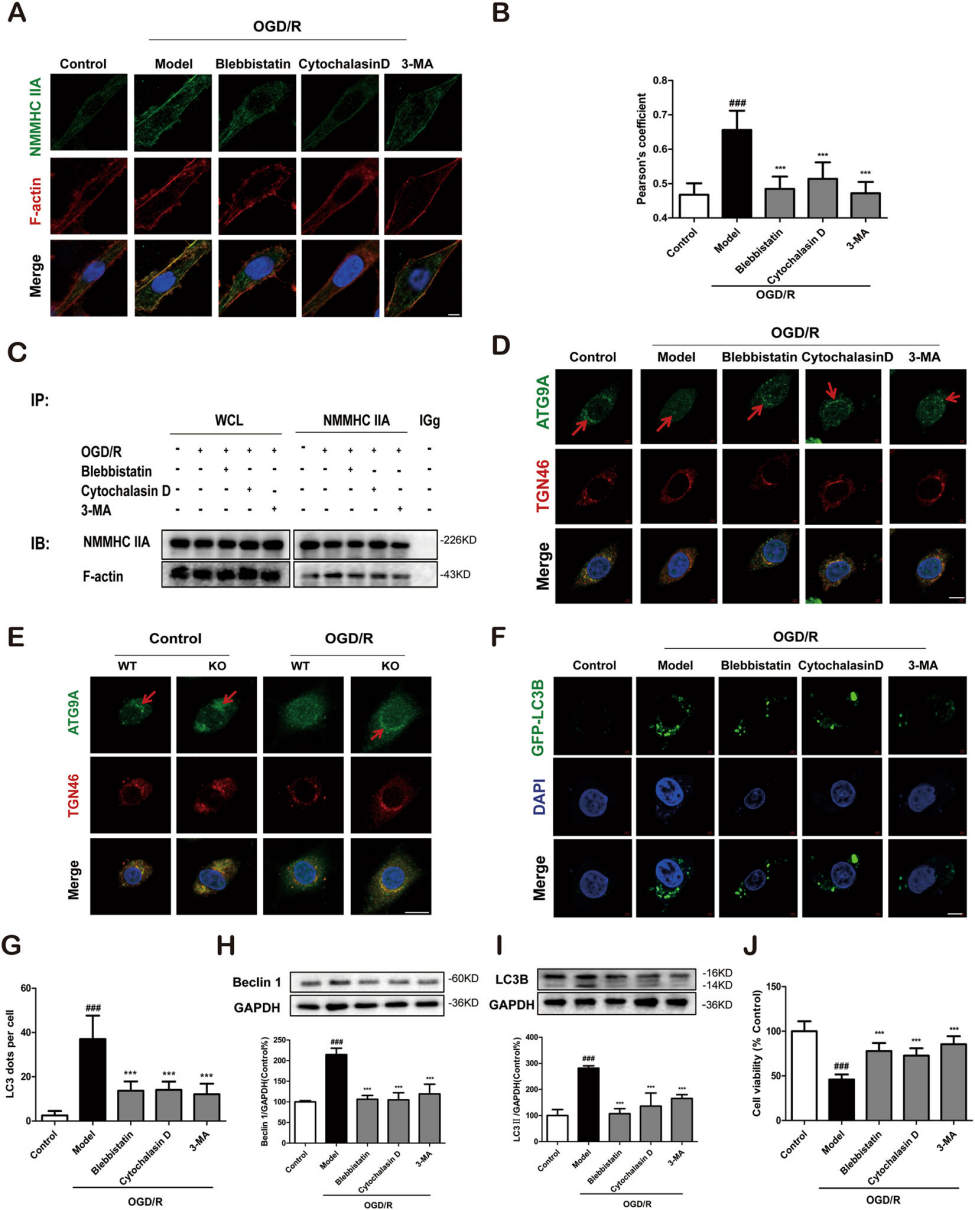
Inhibition of the NMMHC IIA–actin interaction alleviates ATG9A trafficking and neuronal autophagic cell death during OGD/R in PC12 cells. PC12 cells were treated with 6 h of OGD and then reoxygenated for 6 h. **a** Confocal microscopy was used to detect NMMHC IIA (green), F-actin (red), and DAPI (blue). Bar: 2 µm. **b** Colocalization of NMMHC IIA with F-actin was evaluated by Pearson’s coefficients. **c** Co-IP was used to detect protein interactions between actin and NMMHC IIA. **d** ATG9A (green), TGN46 (red), and DAPI (blue) were detected by confocal microscopy. Bar: 2 µm. **e** ATG9A (green), TGN46 (red), and DAPI (blue) were detected by confocal microscopy in NMMHC IIA knockout PC12 cells. Bar: 2 µm. **f**, **g** PC12 cells were transfected with GFP-LC3B plasmid, and EGFP-LC3B puncta was detected after OGD/R treatment. Bar: 2 µm. **h**, **i** Beclin 1 and LC3B expression are depicted by immunoblotting. **j** Cell viability was evaluated by MTT assay upon OGD/R treatment. Results are expressed as the mean ± SD, *n* = 3. ^##^*P* < 0.01 vs. control group; ^**^P < 0.01, ^*^P < 0.05 vs. Model group.

To verify the above hypothesis, blebbistatin and cytochalasin D were used to attenuate the NMMHC IIA–actin interaction induced by OGD/R in PC12 cells. Immunofluorescence and Co-IP analyses showed that blebbistatin and cytochalasin D significantly decreased the NMMHC IIA–actin interaction (Fig. 5a–c; Supplementary Fig. S7A). Next, we detected the effect of inhibiting NMMHC IIA–actin interaction on the transport of ATG9A from TGN by immunofluorescence. As shown in Fig. 5d, ATG9A was enriched in TGN labeled with anti-TGN46 antibody under normal conditions, while ATG9A was redistributed from the TGN to a dispersed peripheral pool in response to OGD/R. Furthermore, OGD/R-induced ATG9A redistribution was blocked by blebbistatin and cytochalasin D. Quantitative results also revealed that blebbistatin and cytochalasin weakened the juxta-nuclear distribution of ATG9A and its colocalization with TGN46 (Supplementary Fig. S7B). In addition, in both normal and OGD/R conditions, more ATG9A was aggregated in NMMHC IIA KO PC12 cells compared with WT cells, due to decreased AGT9A trafficking propelled by NMHHC IIA (Fig. 5e). Pearson correlation coefficient also revealed similar results (Supplementary Fig. S7C). Finally, we investigated the effects of blebbistatin and cytochalasin D on OGD/R-induced autophagy and cell damage. There were fewer EGFP-LC3B puncta in blebbistatin and cytochalasin D groups than in the NC group after OGD/R treatment (Fig. 5f, g). Blebbistatin and cytochalasin D significantly reduced the number of typical autophagosomes and MDC fluorescence intensity (Supplementary Fig. S8). The inhibitors also inhibited autophagy as shown by decreased expression of Beclin 1 and LC3B-II (Fig. 5h, i) and attenuated cell death in response to OGD/R (Fig. 5j). We also demonstrated that blebbistatin, cytochalasin D, and 3-MA increased the expression of Bcl-2 and decreased the level of cleaved caspase-3 and Bax under OGD/R condition in vitro, and I/R in vivo (Supplementary Fig. S9). These results demonstrate that the anti-apoptotic effects of inhibiting NMMHC IIA–actin interaction is via inhibiting autophagy.

**Fig. 6:**
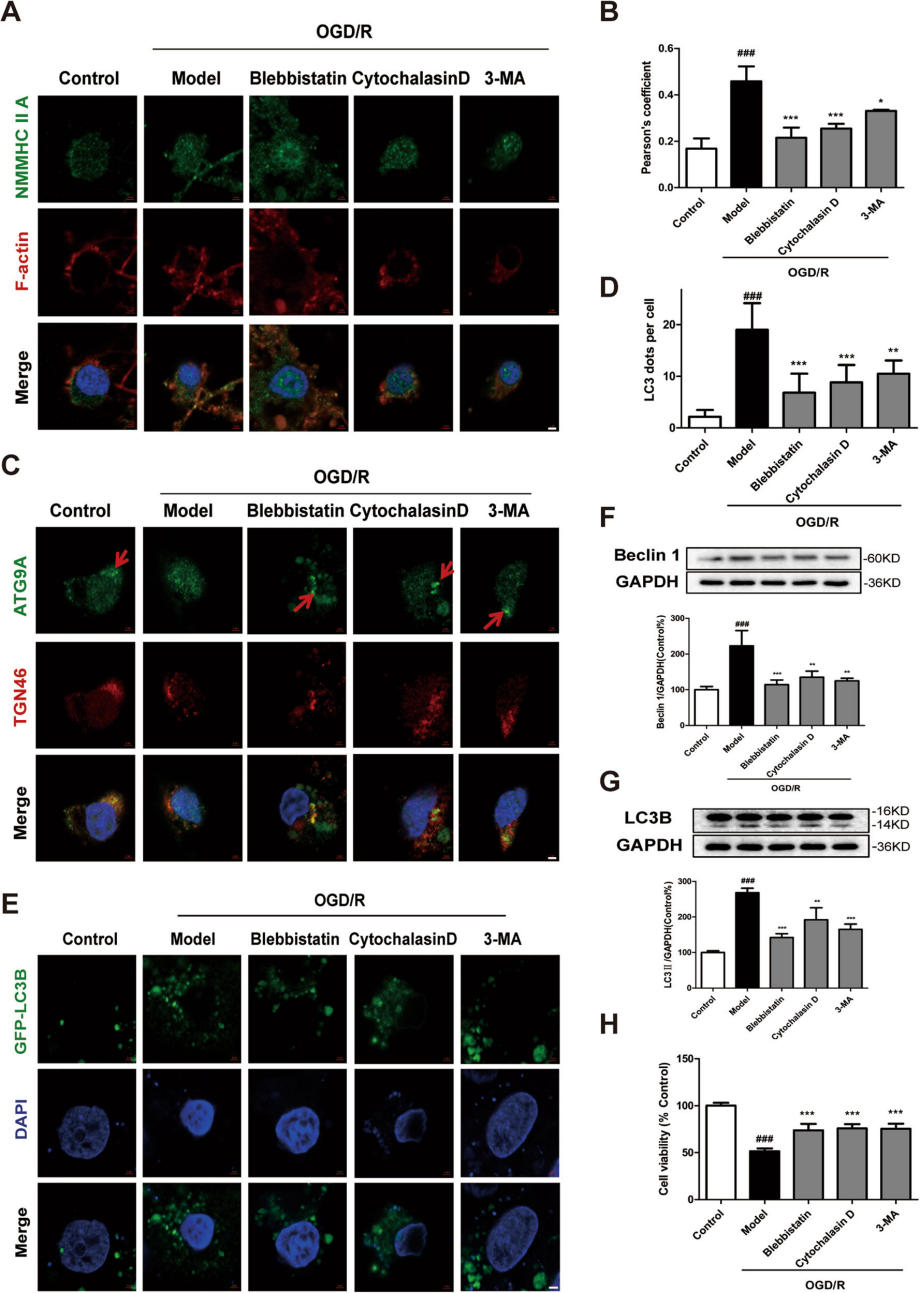
Inhibition of the NMMHC IIA–actin interaction alleviates ATG9A trafficking and neuronal autophagic cell death during OGD/R in primary cortical neurons. Neurons were treated with 6 h of OGD and then reoxygenated for 6 h. **a** Confocal microscopy was used to detect NMMHC IIA (green), F-actin (red), and DAPI (blue). Bar: 2 µm. **b** Colocalization of NMMHC IIA with F-actin was evaluated by Pearson’s coefficients. **c** ATG9A (green), TGN46 (red), and DAPI (blue) were detected by confocal microscopy. Bar: 2 µm. Ble blebbistatin. **d**, **e** Primary cortical neurons were transfected with the GFP-LC3B plasmid, and EGFP-LC3B puncta were detected after OGD/R treatment. Bar: 2 µm. **f**, **g** Beclin and LC3B-II expression are shown by immunoblotting. **h** Cell viability was evaluated by MTT assay upon OGD/R treatment. Results are expressed as the mean ± SD, *n* = 3. ^###^*P* < 0.001 vs. control group; ^*^P < 0.05, ^**^P < 0.01, and ^***^P < 0.001 vs. OGD/R-treated group.

Next, we evaluated the anti-autophagic effect of inhibiting NMHHC IIA–actin interaction in primary cortical neurons under OGD/R. Consistently, blebbistatin, cytochalasin D, or 3-MA treatment inhibited NMMHC IIA–actin interaction (Fig. 6a, b), movement of ATG9A from the TGN (Fig. 6c; Supplementary Fig. S7D), GFP-LC3B puncta (Fig. 6d, e), Beclin 1, and LC3B-II expression (Fig. 6f, g). Moreover, the inhibitors also had neuroprotective effects on the cell viability of cortical neurons (Fig. 6h).

### Blebbistatin alleviates cerebral ischemia/reperfusion injury

**Fig. 7:**
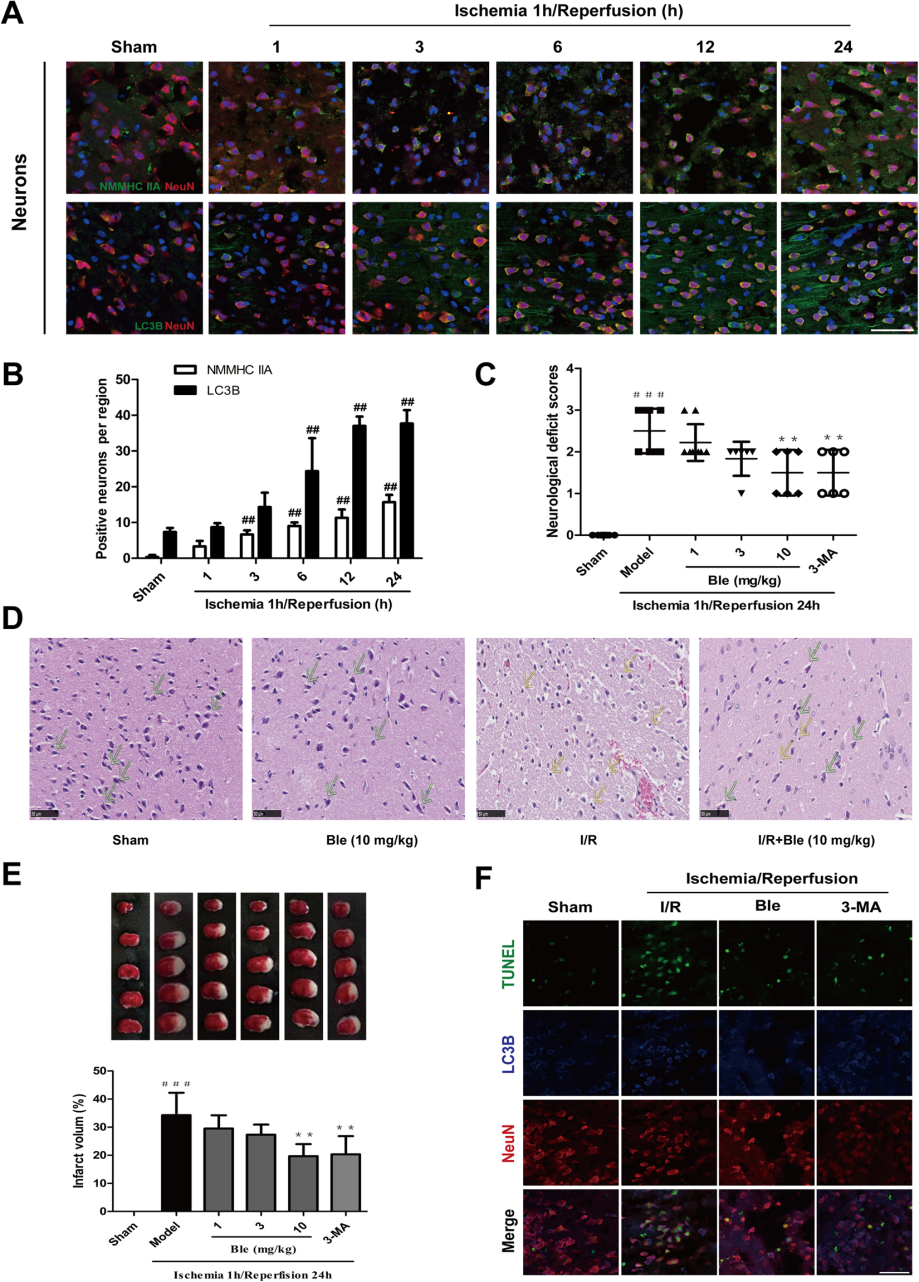
Blebbistatin alleviates cerebral ischemia/reperfusion injury. **a** Mice were treated with 1 h of cerebral ischemia and reperfusion for different time points. Double immunostaining shows the localization of NMMHC IIA (LC3B) in neurons. Bar: 50 µm. **b** Quantification of NMMHC IIA and LC3B-positive neurons. ^##^*P* < 0.01 vs. the sham group. Mice were treated with 1 h of cerebral ischemia and reperfusion for 2 h. **c** Neurological deficit scores in different groups. **d** H&E staining showing the morphological characteristics of mouse brains upon MCAO/R. Bar: 50 µm. **e** Representative TTC-stained brain sections and quantitative analysis of infarct volume in different groups. Results are expressed as the mean ± SD, *n* = 6–11. ^###^*P* < 0.001 *vs*. sham group; ^**^P < 0.01 and ^***^P < 0.001 *vs*. model group. **f** TUNEL staining showing apoptotic neurons in response to MCAO/R. Bar: 50 µm.

The expression of NMMHC IIA and LC3B in neurons was further investigated in ischemic brains after MCAO/R. We found that NMMHC IIA-positive neurons began to increase at 3 h of reperfusion and reached their peak at 24 h (Fig. 7a, b). LC3B-II expression in neurons had similar results (Fig. 7a, b). These results suggest that NMMHC IIA may lead to neuronal autophagy induced by cerebral ischemia–reperfusion. To investigate the function of NMMHC IIA in ischemic attack, we used blebbistatin, which non-specifically suppresses myosin II activity. Twenty-four hours after reperfusion, both neurological deficits and infarct size were reduced in blebbistatin-treated mice compared with the model group (Fig. 7c–e). The 3-MA-treated group had similar results (Fig. 7c–e). In the I/R group, H&E staining showed a large number of shrunken cells with pyknotic nuclei (yellow arrow), which indicated dead cells (Fig. 7d). Notably, the abundance of dead cells decreased, and there were many intact cells (green arrow) in the blebbistatin group (Fig. 7d; Supplementary Fig. S7E). Immunofluorescence showed that blebbistatin and 3-MA treatment decreased apoptotic neurons compared with the I/R group. Moreover, most of the apoptotic neurons were accompanied by autophagy under ischemic attack (red arrow). Blebbistatin also reduced the number of LC3B-positive apoptotic neurons (Fig. 7f; Supplementary Fig. S7F). These results demonstrate that inhibition of NMMHC IIA attenuates neuronal injury, which is associated with inhibition of neuronal autophagic cell death induced by MCAO/R.

### Blebbistatin decreases ATG9A trafficking and neuronal autophagy caused by cerebral ischemia/reperfusion

**Fig. 8:**
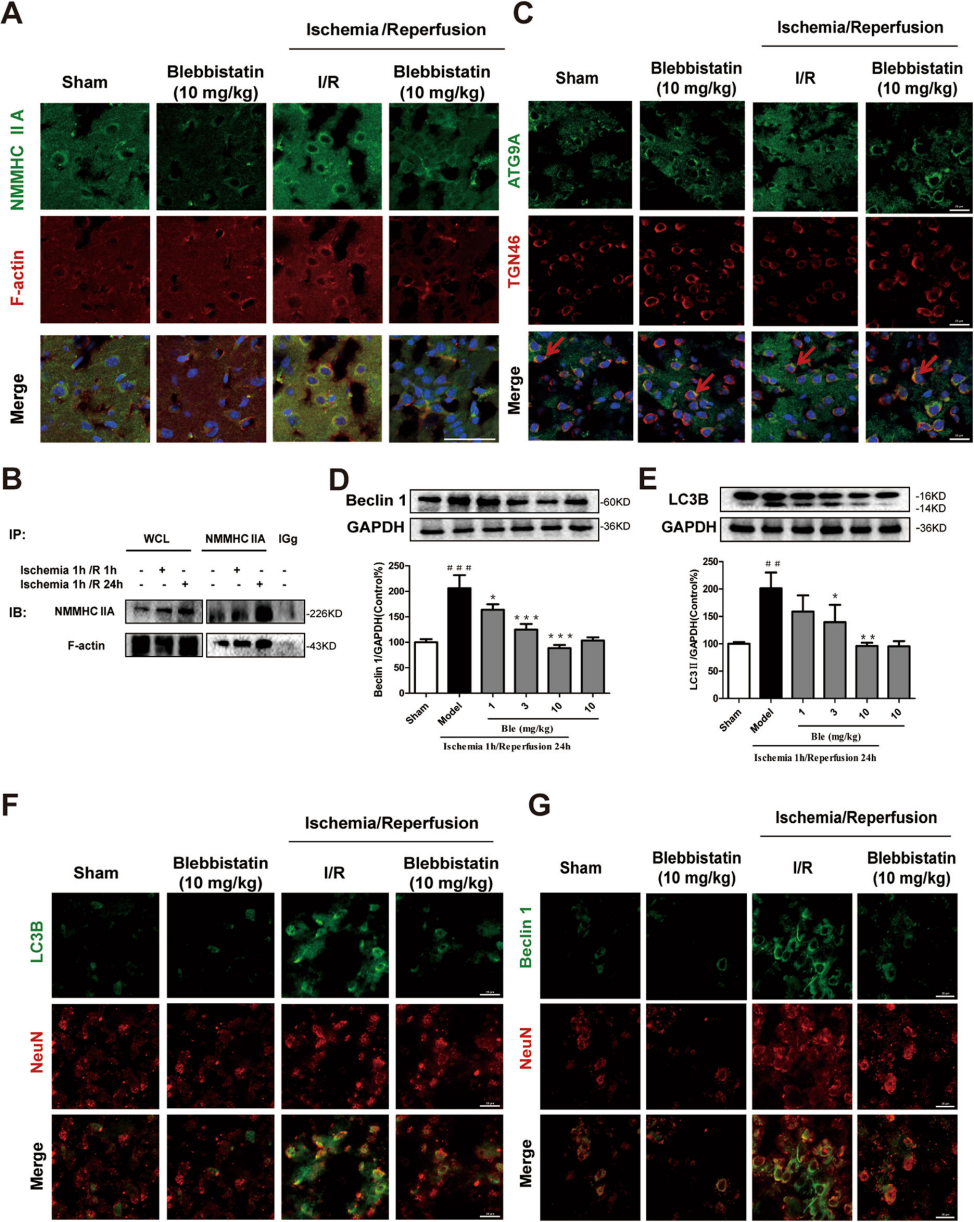
Blebbistatin decreases ATG9A trafficking and neuronal autophagy caused by cerebral ischemia/reperfusion. Mice were treated with 1 h of cerebral ischemia and reperfusion for 2 h. **a** Confocal microscopy was used to detect NMMHC IIA (blue), F-actin (red), and Neun (green). Bar: 50 µm. **b** Co-IP was used to detect protein interactions between actin and NMMHC IIA upon MCAO/R. **C** ATG9A (green), TGN46 (red), and DAPI (blue) were detected by confocal microscopy. Bar: 20 µm. **d**, **e** Beclin and LC3B-II expression are depicted by immunoblotting. Results are were expressed as the mean ± SD, *n* = 3. ^##^*P* < 0.01 and ^###^*P* < 0.001 vs. sham group; ^*^P < 0.05, ^**^P < 0.01, and ^***^P < 0.001 vs. model group. **f**, **g** Then confocal microscope was used to detect Beclin 1/LC3B (green) and Neun (red). Bar: 20 µm.

Next, we examined whether NMMHC IIA influenced ischemia-induced autophagy and the related mechanisms in vivo. As shown in Fig. 8a, b, the interaction between NMMHC IIA and F-actin was increased in ischemia/reperfusion, which was inhibited by blebbistatin treatment. During ischemia/reperfusion, ATG9A was dispersed to the peripheral region, while blebbistatin blocked its peripheral distribution and increased its localization in TGN (Fig. 8c). Moreover, blebbistatin-generated ATG9A retention led to autophagy inhibition, with decreased Beclin 1 and LC3B-II levels (Fig. 8d, e). According to immunostaning results, blebbistatin remarkably reduced Beclin 1 and LC3B fluorescence in neurons caused by ischemia/reperfusion (Fig. 8f, g). These results suggest that NMMHC IIA inhibition exerts protective effects against neuronal autophagy and ischemic injury.

## Discussion

Our study demonstrate that NMMHC IIA inhibition is neuroprotective in cerebral ischemia/reperfusion injury through the coordination of neuronal autophagic cell death. We revealed that NMMHC IIA induces excessive autophagy via interactions with F-actin and ATG9A during OGD/R. We speculated that the contractile force produced by the interaction of NMMHC IIA and F-actin mediated transport of ATG9A with the autophagic membrane, resulting in excessive autophagy and neuronal damage. Furthermore, pharmacological inhibition of NMMHC IIA–F-actin interaction using blebbistatin or cytochalasin D prevented the transposition of ATG9A from the TGN and neuronal autophagic cell death (Supplementary Fig. S10).

NMMHC IIA is widely expressed in eukaryotic cells and interacts with actin filaments to form a contractile unit^26–28^. In the central nervous system (CNS), NMMHC IIA plays an important role. Previous reports revealed that Rho-kinase-dependent activation of NMMHC IIA is essential for neurite outgrowth inhibition produced by repulsive guidance molecules^29^. In the process of H_2_O_2_-induced neuronal apoptosis, NMMHC IIA is required to develop contractile forces and membrane blebbing, and the caspase-3/ROCK1/MLC pathway is involved in the regulation of NMMHC IIA^25^. NMMHC IIA and Annexin 1 are considered as TRPM7 kinase substrates, and TRPM7 kinase modulates OGD/R-induced neuronal apoptosis via annexin 1 carried by NMMHC IIA^30^. These findings indicate the potential role of NMMHC IIA in stroke. However, its particular functions in cerebral ischemia-induced cerebral infarction and neuronal autophagy remain unknown. In our study, we provide the first evidence that NMMHC IIA expression in neurons is increased after cerebral ischemia treatment in vitro and in vivo (Figs. 2a and 7a). Moreover, inhibition of NMMHC IIA by its inhibitor, knockdown or knockout alleviated cerebral ischemia-induced neuronal death (Figs. 1 and 7).

On the other hand, it has been demonstrated that neuronal death is closely related to autophagy. Selective deletion of neuronal Atg7 in mice inhibited hypoxia-induced neuronal autophagy and reduced neuronal death in multiple brain regions^31^. N-acetylserotonin and carnosine inhibited cell death induced by NMDA or oxygen–glucose deprivation in neurons, and their neuroprotective effects result from suppression of autophagy activation under stress conditions^32,33^. Therefore, autophagy in neurons under ischemic attack is believed to be a pattern of cell death that exhibits harmful effects in ischemic stroke. In this study, we found that inhibition of NMMHC IIA in PC12 cells or primary cortical neurons significantly reduced autophagic activation after OGD/R (Fig. 2). In vivo experiments also showed consistent results using blebbistatin (Fig. 8). These findings first present the regulatory role of NMMHC IIA in neuronal autophagy in response to ischemic stress, indicating that NMMHC IIA might induce neuronal autophagic cell death in cerebral ischemia.

Reportedly, ATG9A is a core Atg protein with multiple transmembrane domains^34,35^. In mammalian cells, Atg9A primarily localizes in the TGN and the endosomal system, and cycles between them though vesicle transport. When autophagy occurs, Atg9A is temporarily located on the autophagic membrane. Recent studies demonstrate that membrane trafficking pathways concerned with adaptor/clathrin proteins^36,37^, Rab GTPases^38^, and the retromer complex^39^ are important for both autophagosome formation and ATG9A localization. ATG9A has important physiological functions, and conditional knockout mice of Atg9a in the brain causes axon-specific lesions^40^. Meanwhile, starved cells treated with actin-depolymerizing agents, fail to form the autophagosome structure, revealing the important role of actin in autophagy. In addition, some studies show the colocalization of actin with important autophagy markers, and the trafficking of ATG9A through early and recycling endosomes to the site of autophagosome formation requires actin-binding proteins, such as Annexin A2 and actin nucleators, including Spire1, ARP2/3 and the WASH complex^12,39,41,42^. Furthermore, Atg1-mediated NMMHC II activation is required for the movement of ATG9A transmembrane protein between the TGN and the PAS, which provides a membrane source for autophagosomes formation during starvation^17^. These findings potentially indicate the interaction of ATG9A, actin, and NMMHC IIA; however, there has been no direct evidence reported, particularly in neuronal autophagy. Our results further demonstrated that the interaction between NMMHC IIA and F-actin/ATG9A increased upon OGD/R (Fig. 3). Moreover, NMMHC IIA and actin were well colocalized after OGD/R, and trafficking of ATG9A from the TGN was inhibited after blebbistatin and cytochalasin D treatment, indicating that F-actin is involved in NMMHC IIA-mediated autophagy activation under ischemic conditions (Figs. 5 and 6). Therefore, we postulate that NMMHC IIA might act as a molecular motor interacting with F-actin to promote ATG9A trafficking for autophagosome formation in neurons. These findings provide solid evidence for the importance of NMMHC IIA in ischemic stroke.

The existing structural analysis of NMMHC IIA confirmed that it contains a highly conserved globular head domain, which consists of actin-binding sites^43^. In our study, Co-IP showed that the head domain (aa. 83–764) of NMMHC IIA is essential for NMMHC IIA interaction with F-actin upon OGD/R. Studies have found that the calcium-binding protein S100A4 interacts with the C-terminus of NMMHC IIA. This interaction regulates the adhesion and migration of tumor cells^44^. Gelsolin and C-terminus of NMMHC IIA interact to mediate calcium-regulated collagen phagocytosis^45^. Our results first showed that the tail domain (C-terminal) of NMMHC IIA was necessary for its interaction with ATG9A (Fig. 4). This is of great significance to further study the mechanism of NMMHC IIA involved in autophagy and the development of drugs.

It has been indicated that NMMHC IIA can induce neuronal apoptosis upon cerebral ischemia, by decreasing annexin 1 nuclear translocation^30^. In this study, we found the increasing expression of NMMHC IIA resulted in autophagy activation, concomitant with neuronal apoptosis in vivo and in vitro. Previous studies also confirmed that cells have colocalized apoptosis and autophagy^46,47^. It is reported that the autophagy–apoptosis relationship varies according to different cells and the cerebral area. In the cortex, they are closely related to each other, while in the hippocampus they act independently^47^. Based on these results, we then tested whether the regulation of NMMHC IIA on autophagy led to the antiapoptosis effect of NMMHC IIA. The autophagy inhibitor 3-MA inhibited OGD/R-induced neuronal apoptosis, suggesting the involvement of autophagy-mediated apoptosis. Generally, NMMHC IIA may be an important regulatory protein to control autophagy and apoptosis.

Many autophagy-related genes and proteins are altered in numerous diseases, which provides novel targets for therapeutic intervention. Our findings identified NMMHC IIA as a critical regulator of cerebral ischemia-induced neuronal autophagy and provided mechanistic insights into cerebral ischemia progression that can be exploited for pharmacological intervention. As a myosin II inhibitor, blebbistatin has proven highly useful in a broad range of research areas, including neuroscience, cardiac physiology, and cancer research. In addition to research applications, blebbistatin also possesses great potential as a lead compound for treating various myosin II-related diseases. In our study, blebbistatin attenuated neuronal injury in cerebral ischemia both in vivo and in vitro. However, application of blebbistatin requires extra care as the compound has several nonspecific adverse effects including cytotoxicity, phototoxicity, and structural instability, which may mask possible myosin-specific effects. To overcome these hurdles, adeno-associated virus (AAV) vectors expressing shRNAs targeting the mouse MYH9 gene can be developed. A number of factors make AAV an ideal gene therapy for the CNS. For example, AAV exhibits a strong preference for neuronal transduction, supports very long-lasting expression from a single delivery, and exhibits no pathogenicity^48,49^. In addition, natural products targeting NMMHC IIA will be screened for treatment of stroke because of their potential complementary therapy and fewer adverse effects.

In conclusion, we show that autophagy induction by NMMHC IIA is involved in cerebral ischemia/reperfusion-induced neuronal cell death, which could be attributed to interactions with F-actin and transport ATG9A from TGN. Further experiments in NMMHC IIA knockout mice and related signaling pathways regulating NMMHC IIA–actin interactions induced by cerebral ischemia will be performed in the future.

## Materials and methods

### Antibodies and reagents

Antibodies against Beclin 1 (ab55878), LC3B (ab192890), NMMHC IIA (ab75590), F-actin (ab205), Atg9A (ab108338), MAP2 (ab11267), TGN46 (ab2809), NeuN (ab104224), and cytochalasin D (ab143484) were obtained from Abcam (Cambridge, UK). L-glutamine (25030–081), neurobasal medium (21103049), B27 supplement (50×, minus antioxidants, 10889–038), soybean trypsin inhibitor (17075029), Alexa Fluor 594 goat anti-mouse antibody (A11032) and Alexa Fluor 488 donkey anti-rabbit antibody (A21206) were obtained from Thermo Fisher Scientific (San Jose, CA, USA). Cytosine arabinoside (C6645), poly-L-lysine (P1274), D-( + )-glucose (G7021), and 3-MA (M9281) were from Sigma-Aldrich (St. Louis, MO, USA). Blebbistatin (S7099) was obtained from Selleck Chemicals (Houston, TX, USA). ExFect Transfection Reagent (T101–01) was purchased from Vazyme Biotech Co., Ltd (Nanjing, China).

### Animals and treatment

Male specified pathogen free (SPF) C57BL/6J mice weighing 18–22 g were obtained from the Reference Animal Research Centre of Yangzhou University (Yangzhou, China; certificate no SCXK 2017–0001). All experimental protocols were performed according to the National Institutes of Health (NIH) guidelines, and the research was approved by the Institutional Animal Care and Use Committee of the Animal Ethics Committee of the School of Chinese Materia Medica, China Pharmaceutical University.

### Cell culture

Highly differentiated PC12 cells were obtained from the Shanghai Institute of Cell Biology (Shanghai, China). Differentiated PC12 cells were cultured in DMEM supplemented with 10% fetal bovine serum at 37 °C in a humidified cell culture incubator in 5% CO_2_/95% air.

### Primary cortical neuron cultures

Cortical neurons were cultured from Sprague–Dawley rats as described previously^25^. Pregnant Sprague–Dawley rats were purchased from the Reference Animal Research Centre of Yangzhou University (Yangzhou, China; certificate no SCXK 2017–0001). Briefly, cerebral cortices were removed from embryos at 16–17 days, stripped of meninges and blood vessels, and minced. Tissues were dissociated through trypsin digestion, two successive trituration and sedimentation steps in isolation buffer. Cells were centrifuged (1000 r/min, 5 min) and then resuspended in neurobasal medium containing 2% B27 supplement, 5% fetal bovine serum, 10 mM HEPES buffer solution and 1 mM L-glutamine, and then the neurons were plated onto PLL (0.5 mg/mL)-coated 96-well plates at a density of 1 × 10^6^ cells/mL. Neurons were cultured at 37 °C in a humidified 5% CO_2_ incubator, and half of the culture medium was changed every 3 days using neurobasal medium containing B27 and L-glutamine.

### In vivo cerebral ischemia/reperfusion model

The MCAO/R model was prepared in mice as described previously^50^. Cerebral ischemia was induced by intraluminal occlusion of the right middle cerebral artery using a silicone rubber-coated 6–0 nylon monofilament. To confirm the cerebral artery blood flow, a laser Doppler flow meter (LDF; FLPI2, Moor, UK) was used. Approximately 1 h after occlusion, the suture was withdrawn to allow reperfusion for 24 h. Neurological deficit was examined using Longa’s method^50^. Infarct size was determined by staining with TTC and analyzed using ImageJ software. The infarct areas on each slice were summed and multiplied by slice thickness to give the infarct volume. Infarct volume was expressed as a percentage of infarction per ipsilateral hemisphere. Blebbistatin and 3-MA were injected immediately before reperfusion, 1 h after MCAO. The 66 mice were divided randomly into six groups (*n* = 11 in each group): sham operated (Sham), I/R, I/R + Blebbistatin (1, 3, 10 mg/kg), and the I/R + 3-MA (300 μg/kg). Blebbistatin was dissolved in saline solution containing 5% ethanol for injection, and the Sham group was administered vehicle.

### Oxygen–glucose deprivation/reperfusion (OGD/R) and drug treatments

Blebbistatin, cytochalasin D, and 3-MA were dissolved in dimethyl sulfoxide (DMSO) and diluted in DMEM culture medium without glucose at various concentrations (Blebbistatin and cytochalasin: 1 µM, 3-MA: 3 mM). The final DMSO concentration is 0.1% (v/v). After cells were treated with drugs, OGD/R was induced in cells for 6 h in a hypoxia chamber in DMEM culture medium without glucose in an atmosphere of 5% CO_2_, 94% N_2_, and 1% O_2_ followed by culture under normoxic conditions, as described previously^50^.

### Haematoxylin and eosin (H&E) staining

Histomorphological analysis was performed by H&E staining. Briefly, brain slices were put into haematoxylin and eosin solution, redehydrated in gradient ethanol solution again, treated with dimethylbenzene, and covered with coverslips. A digital pathological section scanner (Hamamatsu, Japan) was used to screen pathological images. NDPView2 software was used to analyze these images.

### Cell viability

MTT assay was used to detect cell viability. After treatment of cells, medium was replaced with 100 µL culture medium containing 5 mg/mL MTT solution. Four hours after incubation at 37 °C, the reaction solution was removed, and 150 µL DMSO was added to each well. A microplate reader (Epoch, Bio Tek, Winooski, VT, USA) was used to record the absorbance with dual waves at 570 and 650 nm after 10 min of shaking.

### SiRNA transfection and plasmid

Small interfering RNAs (siRNAs) against NMMHC IIA (sense: 5′-GAGACAAUGGAGGCCAUGA-3′, and antisense: 3′-UCAUGGCCUCCAUUGUCUC-5′) were synthesized to knockdown NMMHC IIA by Biomics Biotech (Nantong, China). Plasmids of NMMHC IIA (EX-T1335-M98) and LC3B (S8469–1A) were purchased from the FulenGen Company (Guangzhou, China). Transfection of NMMHC IIA siRNA or plasmids in cells was performed using ExFect Transfection Reagent according to the instructions. After transfection for 48 h, cells were harvested for subsequent experiments.

### Myh9 gene knockout using the CRISPR/Cas9 system

The CRISPR/Cas9 system is an adaptive immune defence mechanism used by bacteria and archaea to degrade foreign genetic material. It can also be used for other functions, including genomic engineering for mammalian systems, such as gene knockout^51,52^. The Myh9 CRISPR/Cas9 knockout plasmid was obtained from Santa Cruz Biotechnology (Dallas, TX, USA). ExFect Transfection Reagent was used to transfect the plasmid. After incubation for 48 h, cells transfected with CRISPR/Cas9 KO plasmid were sorted by detection of the green fluorescent protein (GFP) using a BD FACSAria III cell sorter (BD Biosciences, San Jose, CA, USA). Three weeks later, western blotting was used to screen myh9 knockout cells.

### In vivo and in vitro immunofluorescence

After perfusion with PBS and 4% paraformaldehyde for 3 min, brain tissues were removed and placed into 4% paraformaldehyde at 4 °C. After 1 day, brain tissue was dehydrated using 40% sucrose for 5 days, embedded in OTC, and frozen at −70 °C. Brain tissues were sectioned into slices of 10-µm thickness using a cryotome (Leica, Mannheim, Germany) and then placed on adhesion microscope slides (Citoglas, China). Brain sections or cultured neurons were fixed in 4% paraformaldehyde, permeabilized with 0.3% Triton X-100 in PBS, blocked with 5% normal donkey serum, and incubated overnight at 4 °C with specific primary antibodies. Then, sections were incubated with corresponding secondary antibodies at room temperature. DAPI was used to stain nuclei. The immunofluorescence TUNEL assay was performed according to the instructions of the manufacturer. Fluorescent images were observed under confocal laser scanning microscopy.

### Transfection of cells with fluorescence LC3 adenoviral vectors

The mRFP-GFP-LC3 adenoviral vectors (Ad-mRFP-GFP-LC3) were provided by Genomeditech Co. Ltd. Dissection of the autophagic flux process by a novel reporter protein, tandem fluorescent-tagged LC3. The Ad was transfected into PC12 cells according to the manufacturer’s protocol. Observation of autophagic flux was determined after fluorescent staining by evaluating the number of GFP and mRFP puncta.

### Proximity ligation assays (PLA)

To demonstrate a complex between native ATG9A and NMMHC IIA in PC12 cells, a PLA kit (Sigma-Aldrich) was used as per the manufacturer protocols. Primary antibodies to ATG9A and NMMHC IIA were used, and secondary antibodies were conjugated to oligonucleotides for ligation and subsequent rolling circle amplification.

### Transmission electron microscopy (TEM)

Cell suspensions were centrifuged, resuspended in fixative in a Eppendorf tube, spun down again to form a pellet, and stored overnight at 4 °C. Cells were then postfixed in 1% osmium tetroxide in 0.10 M phosphate buffer, dehydrated in a graded series of ethanol (30, 50, 70, 80, 90, and 100%), infiltrated, and embedded in an “Epon-Araldite” mixture. 75-nm sections were stained with uranyl acetate and bismuth subnitrite, and then examined in a transmission electron microscope (JEM-1010, JEOL Ltd, Tokyo, Japan).

### Monodansylcadaverine (MDC) staining

PC12 cells were incubated in PBS with 100 µmol/L MDC (Sigma-Aldrich Company, St. Louis, MO, USA) solution for 30 min at 37 °C in the dark. After being washed with PBS, they were examined under an Olympus fluorescence microscope with 20 × objective lens magnification.

### Western blot analysis

Ischemic penumbra of the brain tissues (Supplemental Fig. S7G) and cells were lysed in RIPA buffer supplemented with protease inhibitor cocktail and used for western blotting as described previously^22^. After centrifuging at 12,000 rpm for 10 min at 4 °C, a bicinchoninic acid (BCA) protein assay kit (Bi yuntian Biotech. Co., Ltd., China) was used to determine the protein concentration of the supernatant. The supernatant was diluted by loading buffer to 1 μg/μL and then heated at 100 °C for 5 min. Proteins (20 μg/well) were separated by SDA-PAGE and transferred to a PVDF membrane. Blots were blocked for 2 h and incubated with specific primary antibodies and secondary antibodies. Images were detected with ECL and imaged using the Gel Imaging System (BioRad, Hercules, CA, USA).

### Immunoprecipitation

Briefly, 30 µL Protein A/G PLUS-Agarose was washed with RIPA buffer three times, and 2 µg antibodies were added to the agarose solution and incubated at 4 °C overnight on a rotator. The prepared antibody-agarose complex was added to 1 mL of whole-cell lysate (1 µg/µL) and incubated for 6 h at 4 °C, followed by washing three times with RIPA buffer, adding 2 × SDS gel loading buffer and boiling for 5 min. Proteins were detected by western blot analysis.

### Statistical analysis

All data are expressed as the means ± SD from at least three independent experiments. Data were analyzed by Student’s *t* test for two group comparisons or one-way analysis of variance (ANOVA) followed by Dunnett’s post hoc test for multiple comparisons using Graph Pad Prism 6.0 (Graph Pad Software, La Jolla, CA, USA). Differences were considered significant with a *P*-value<0.05.

## Supplementary information

Supplementary Figure Legends

Supplementary Figure 1

Supplementary Figure 2

Supplementary Figure 3

Supplementary Figure 4

Supplementary Figure 5

Supplementary Figure 6

Supplementary Figure 7

Supplementary Figure 8

Supplementary Figure 9

Supplementary Figure 10
