# Supplementary material for: NMMHC IIA triggers neuronal autophagic cell death by promoting F-actin-dependent ATG9A trafficking in cerebral ischemia/reperfusion
Source: Cell Death Dis. 2020 Jun 8;11(6):428. doi: 10.1038/s41419-020-2639-1 (PMC7280511; doi:10.1038/s41419-020-2639-1)
Supplement: Supplementary file 1 — Supplementary Figure Legends [file 41419_2020_2639_MOESM1_ESM.docx]

**Figure legends**

**Fig. S1 Expression of NMMHC IIA in transfected cells.** A Primary cortical neurons were exposed to OGD/R, then the expression of MAP2 were determined by confocal microscope. Bar: 40 µm. B Primary cortical neurons were transfected with MYH9-siRNA for 48 h, followed by detection of NMMHC IIA expression by western blotting. C MYH9 knockout PC12 cells were established by CRISPR/Cas9 system. Cell lysates from single cell colonies 1, 2, and 3, or WT cells were analyzed by western blotting using NMMHC IIA antibody. D, E PC12 cells were transfected with siRNAs against MYH9 or MYH9 overexpression plasmid. 48 h after transfection, the expression of NMMHC IIA was detected by western blotting. F MYH9 gene was deleted by CRISPR/Cas9 system, and then the PC12 cells were transfected with myh9 plasmid. All data are presented as the means±SD of 3 independent experiments. ^##^P<0.01, ^###^P<0.001.

**Fig. S2 Inhibition of autophagy could significantly improve OGD/R-induced cell damage.** A, B Knockdown of Beclin 1 (A) and Atg7 (B) by RNAi in PC12 cells. C PC12 cells were transfected with siRNAs and cell viability was evaluated by MTT after OGD/R treatment. #1,#2,#3 stand for three different siRNA sequences targeting Beclin 1 and Atg7 respectively. ^###^P<0.001 vs. NC group; ^*^P<0.05 and ^**^P<0.01 vs. OGD/R-treated group.

**Fig. S3 Expression of NMMHC IIB, NMMHC IIC and Beclin 1 in primary cortical neurons under OGD/R.** Primary cortical neurons were exposed to OGD/R, then the expression of NMMHC IIB (A), NMMHC IIC (B) and Beclin 1 (C) at different time points of reoxygenation were determined by western blot. PC12 cells were exposed to OGD/R, then the expression of NMMHC IIA (D), NMMHC IIB (E), NMMHC IIC (F), Beclin 1 (G) and LC3B (H) at different time points of reoxygenation were determined by western blot. In Fig. S3G and H, GAPDH, LC3 and Beclin-1 were probed on the same blot. The data are represented as means±SD of 3 individual experiments. NS, no significance. ^##^P<0.01, ^###^P<0.001 vs. the control group.

**Fig. S4 MYH9-siRNA reduced autophagy expression in PC12 Cells.** A, B PC12 cells were transfected with siRNAs against NMMHC IIA or negative control sequence as described in experimental procedures. 48 h after transfection, then the cells were subjected to OGD/R, the expression of Beclin 1 and LC3B determined by western blotting analysis. C, D PC12 cells were co-transfected with GFP-LC3B plasmid and MYH9 siRNA, EGFP-LC3B puncta was detect after OGD/R. Bar: 2 µm. All data are presented as the mean±SD of 3 independent experiments. ^##^P<0.01, ^###^P<0.001 vs. the negative control group; ^***^P<0.001 vs. OGD/R-treated group.

**Fig. S5 MYH9 overexpression plasmid increased Beclin 1 and LC3B expression in MYH9 knockout PC12 cells.** MYH9 gene was deleted by CRISPR/Cas9 system, and then the PC12 cells were transfected with MYH9 plasmid. A, B The expression of NMMHC IIA, Beclin 1 and LC3B were detected by western blot after 48 h transfection. All data are presented as the means±SD of 3 independent experiments. ^##^P<0.01, ^###^P<0.001.

**Fig. S6 Inhibition of the NMMHC IIA-actin interaction alleviates ATG9A trafficking and neuronal autophagic cell death during OGD/R in primary cortical neurons.** Confocal micrographs of PC12 cells in the presence or absence of OGD/R by the PLA reaction to show the NMMHC IIA-ATG9A interaction (red signal) and DAPI (blue) staining. Scale bar, 50 μm.

**Fig. S7 Quantitative data of immunofluorescent results.** A Quantitative analyses for NMMHC IIA-actin interaction. B Colocalization of ATG9A with TGN46 was evaluated by Pearson's coefﬁcients in PC12 cells. C Colocalization of ATG9A with TGN46 was evaluated by Pearson's coefﬁcients in NMMHC IIA knockout PC12 cells. D Colocalization of ATG9A with TGN46 was evaluated by Pearson's coefﬁcients in cortical neurons. E Statistical analysis of intact cells in the ischemic region. F Quantitative assessment of TUNEL/NeuN-positive cells. G A schematic drawing of coronal rat brain slice, the square fields represent observed regions. All data are presented as the mean±SD of 3 independent experiments. ^##^P<0.01 vs. control group; ^**^P<0.01 vs. OGD/R-treated group.

**Fig. S8 Inbibition of NMMHC IIA-actin interaction attenuated autophagosome formation and MDC fluorescence.** PC12 cells were treated with 1 µM blebbistatin and 1 µM cytochalasin D during OGD/R. 3-MA (3 mM) was treated as positive control. A Electron microscopic images showed double-membrane vacuolar structures in PC12 cells. Bar: 2 µm. B Autophagy of neurons was assessed by 50 μM MDC stain. Autophagic vacuoles were labeled by MDC staining. Bar: 300 µm. All data are presented as the mean±SD of 3 independent experiments. ^###^P<0.001 vs. control group; ^**^P<0.01 and ^***^P<0.001 vs. OGD/R-treated group.

**Fig. S9 Treatment of blebbistatin, cytochalasin D and 3-MA decreased the expression of apoptotic key proteins.** A, B, C PC12 cells were subjected to OGD/R, then the expression of bax, Bcl-2 and cleaved Caspase-3 were determined by western blot. D, E, F Immunoblots of bax, Bcl-2 and cleaved Caspase-3 from total cell lysates of cortical tissue harvested 24 h after I/R. GAPDH was used as an control. All data are presented as the mean±SD of 3 independent experiments. ^###^P<0.001 vs. control group; ^*^P<0.01, ^**^P<0.01 and ^***^P<0.001 vs. model group.

**Fig. S10 Schematic model demonstrating that NMMHC IIA induces neuronal autophagic death via interactions with F-actin and ATG9A in cerebral ischemia/reperfusion.** During cerebral ischemia/reperfusion injury, NMMHC IIA-actin interaction increases, which could provide the force to deliver Atg9A membranes to induce neuronal autophagic cell death. Blebbistatin inhibits NMMHC IIA-actin interaction, resulting in decreased Atg9A trafficking and autophagy activation. Therefore, neuronal autophagic cell death was decreased.
