# Supplementary figures and images for: NMMHC IIA triggers neuronal autophagic cell death by promoting F-actin-dependent ATG9A trafficking in cerebral ischemia/reperfusion
Source: Cell Death Dis. 2020 Jun 8;11(6):428. doi: 10.1038/s41419-020-2639-1 (PMC7280511; doi:10.1038/s41419-020-2639-1)

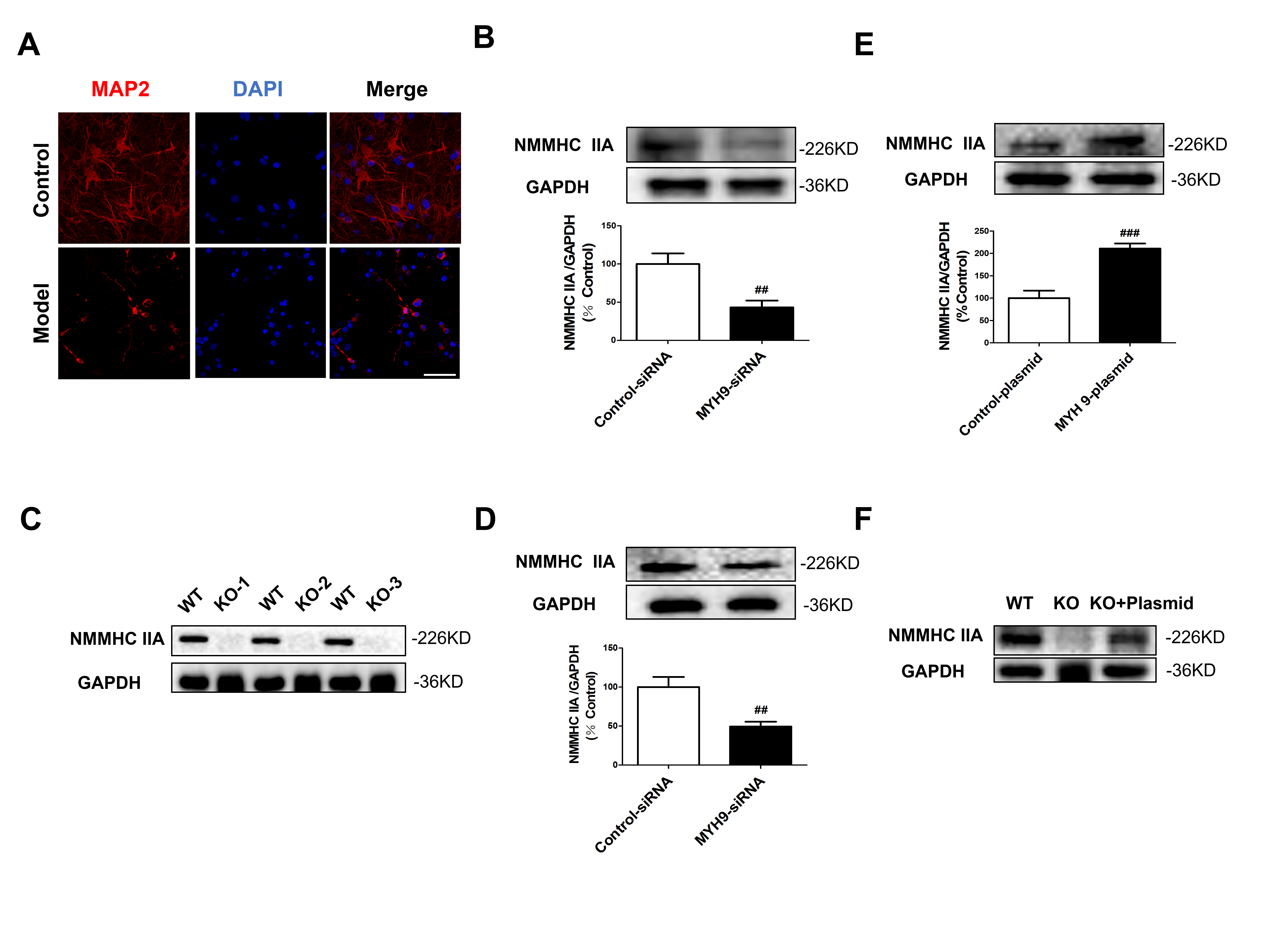

Supplement: Supplementary file 2 — Supplementary Figure 1 [file 41419_2020_2639_MOESM2_ESM.tif]

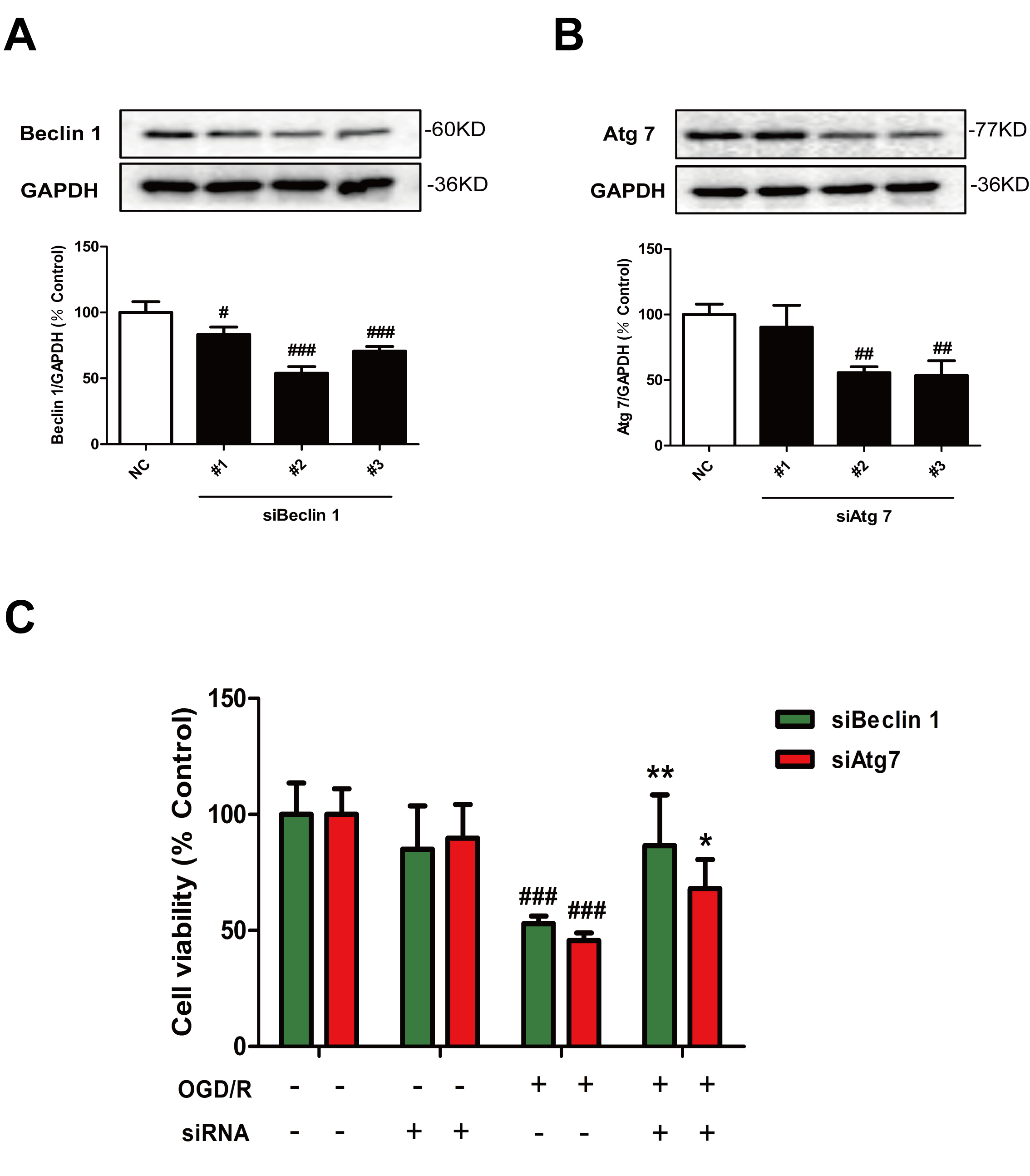

Supplement: Supplementary file 3 — Supplementary Figure 2 [file 41419_2020_2639_MOESM3_ESM.tif]

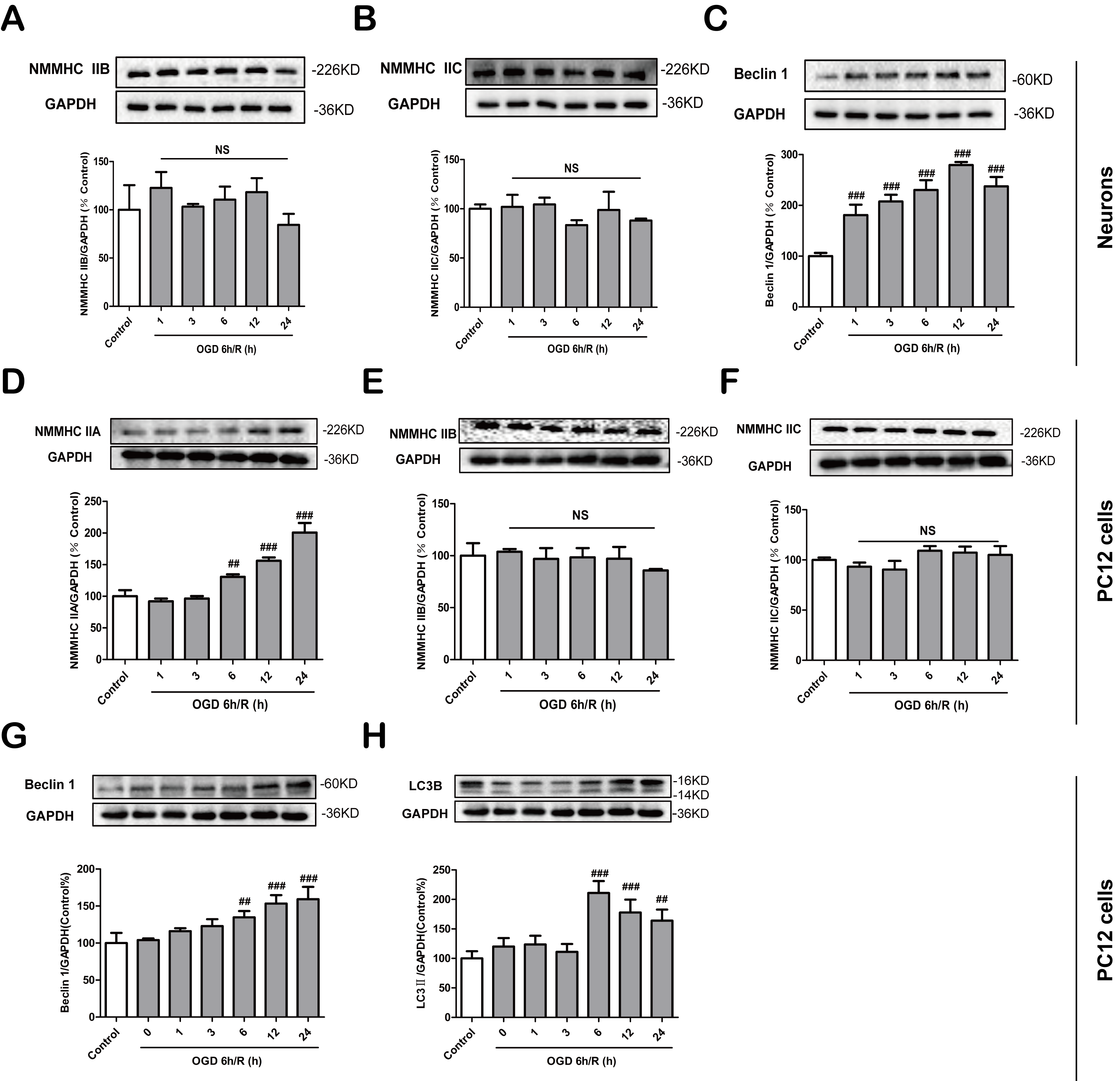

Supplement: Supplementary file 4 — Supplementary Figure 3 [file 41419_2020_2639_MOESM4_ESM.tif]

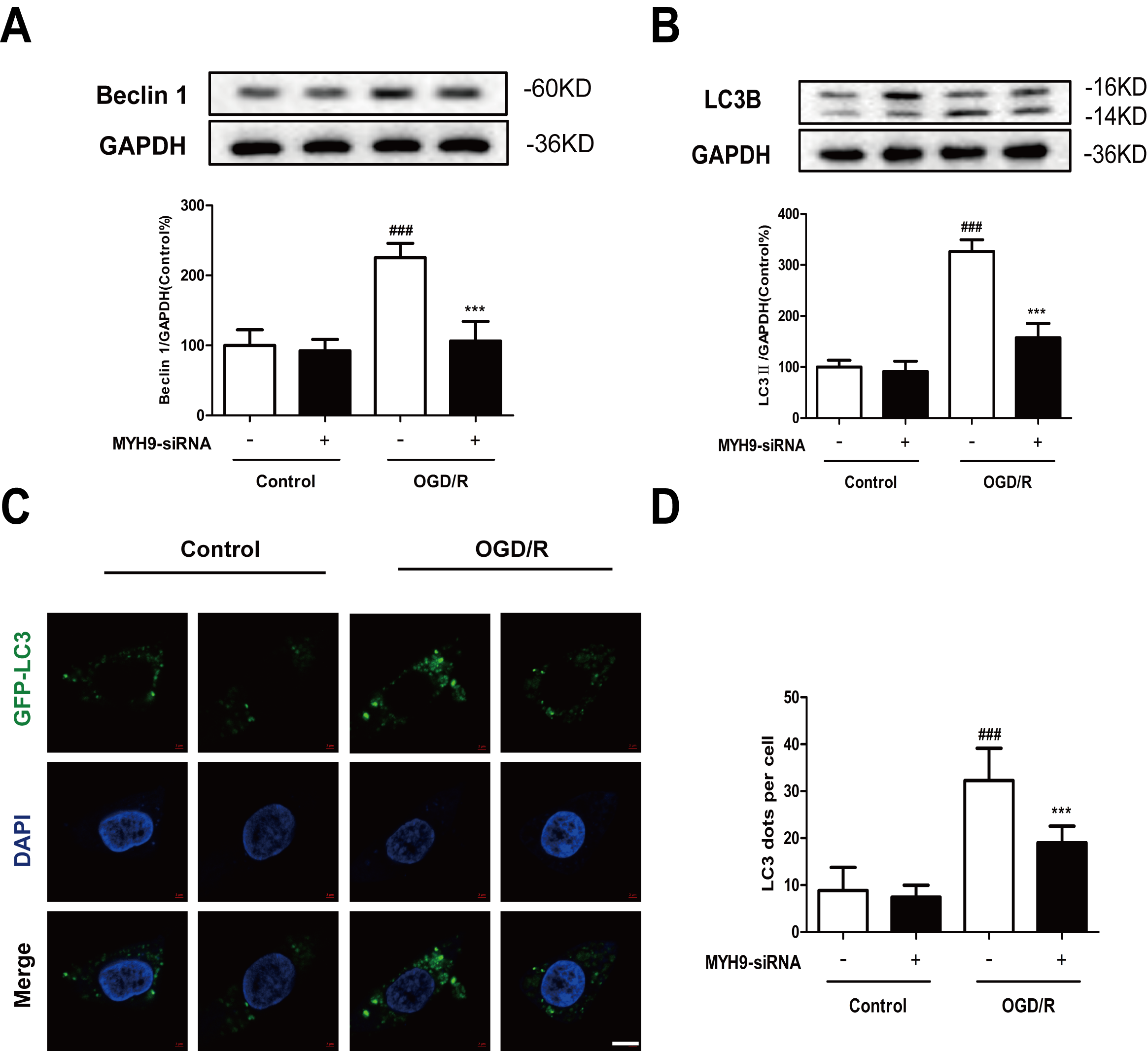

Supplement: Supplementary file 5 — Supplementary Figure 4 [file 41419_2020_2639_MOESM5_ESM.tif]

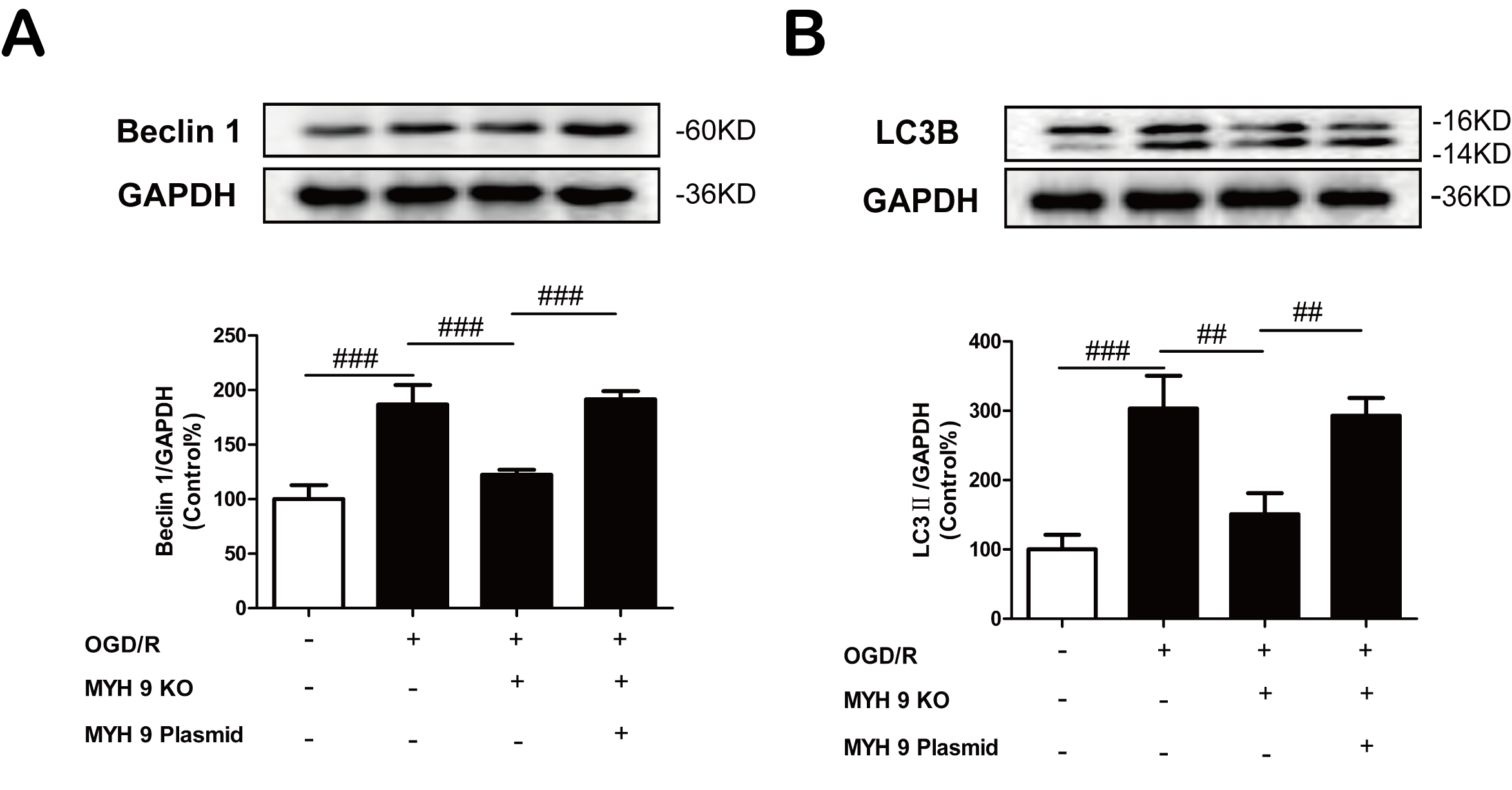

Supplement: Supplementary file 6 — Supplementary Figure 5 [file 41419_2020_2639_MOESM6_ESM.tif]

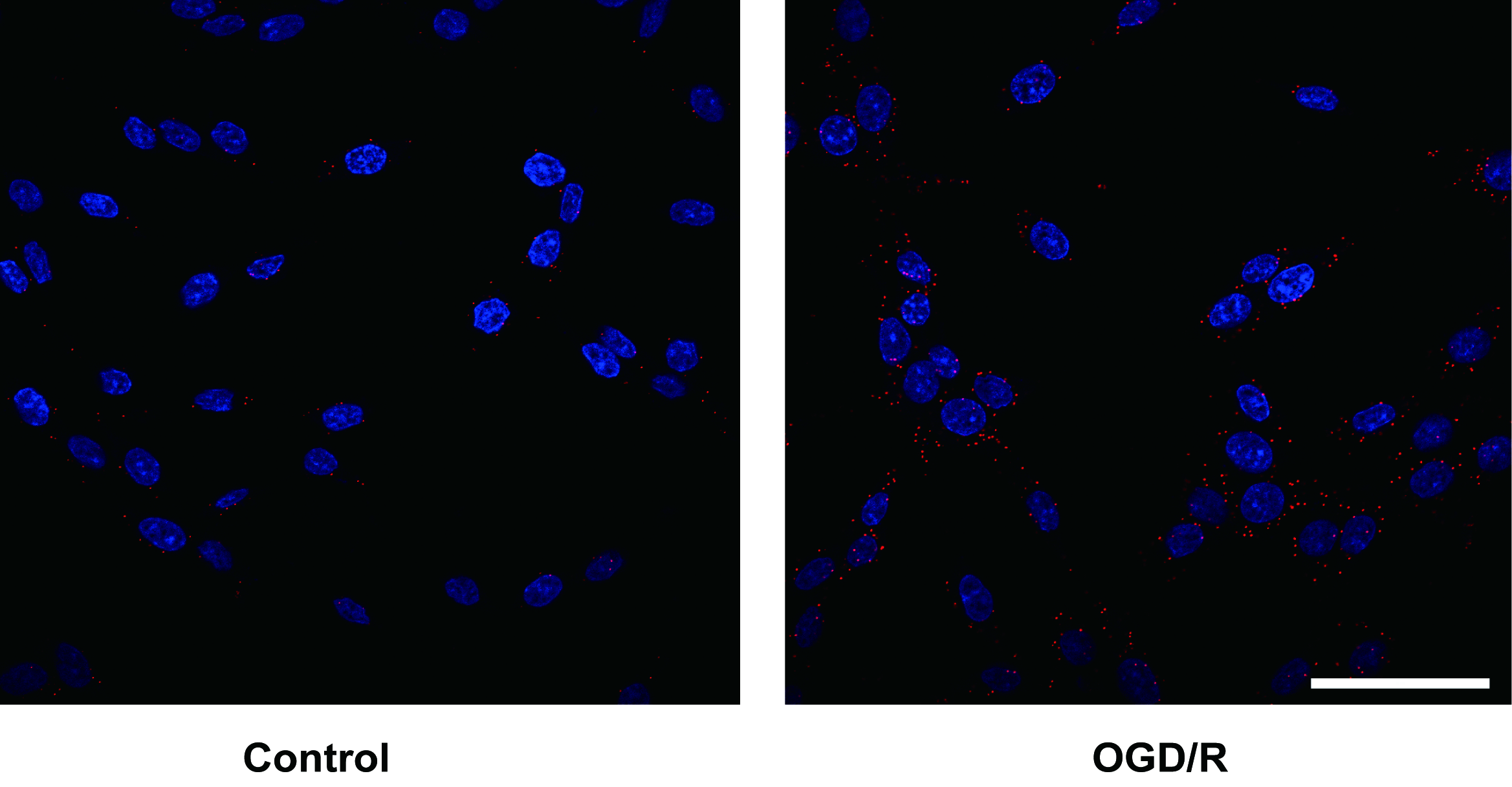

Supplement: Supplementary file 7 — Supplementary Figure 6 [file 41419_2020_2639_MOESM7_ESM.tif]

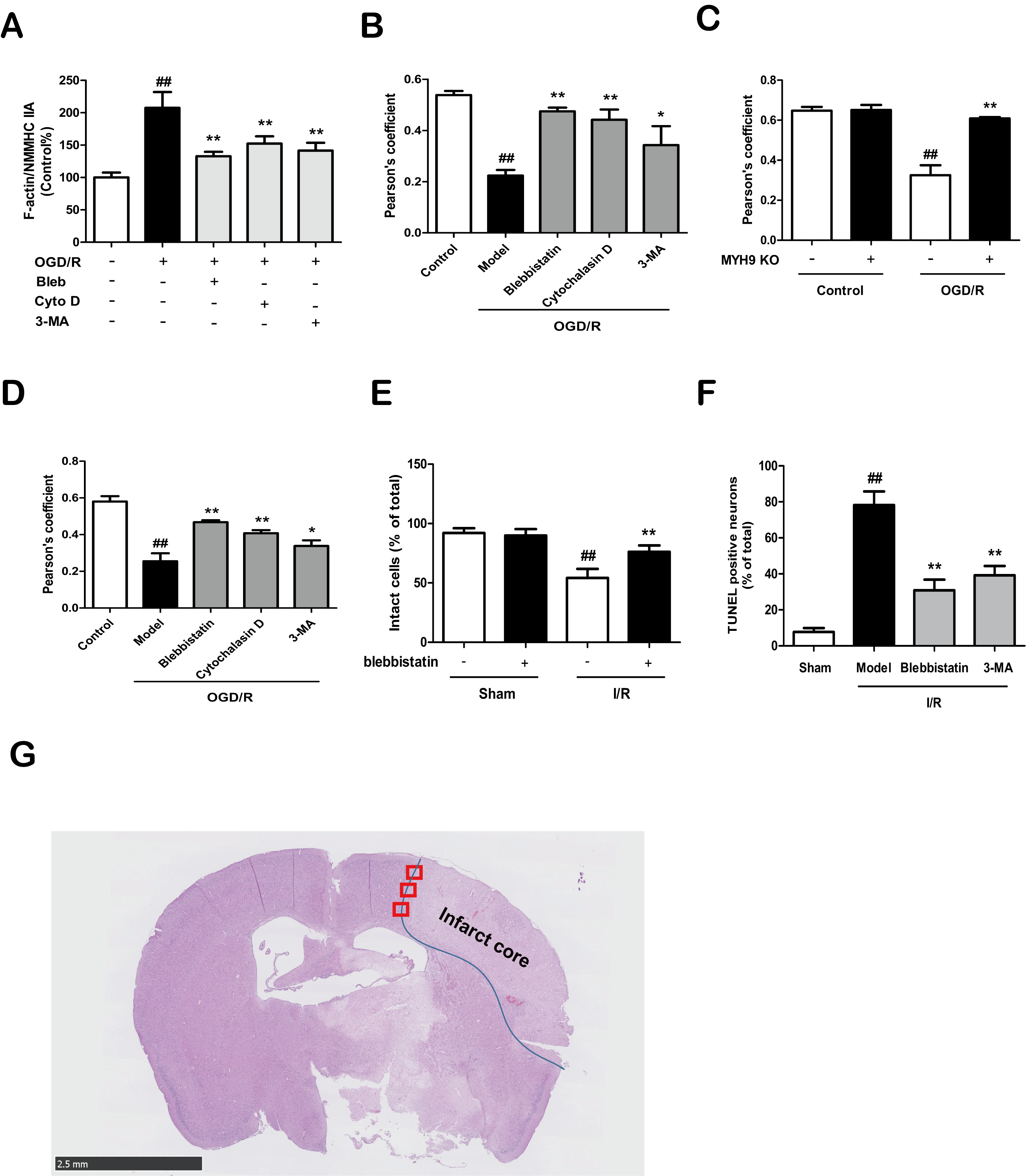

Supplement: Supplementary file 8 — Supplementary Figure 7 [file 41419_2020_2639_MOESM8_ESM.tif]

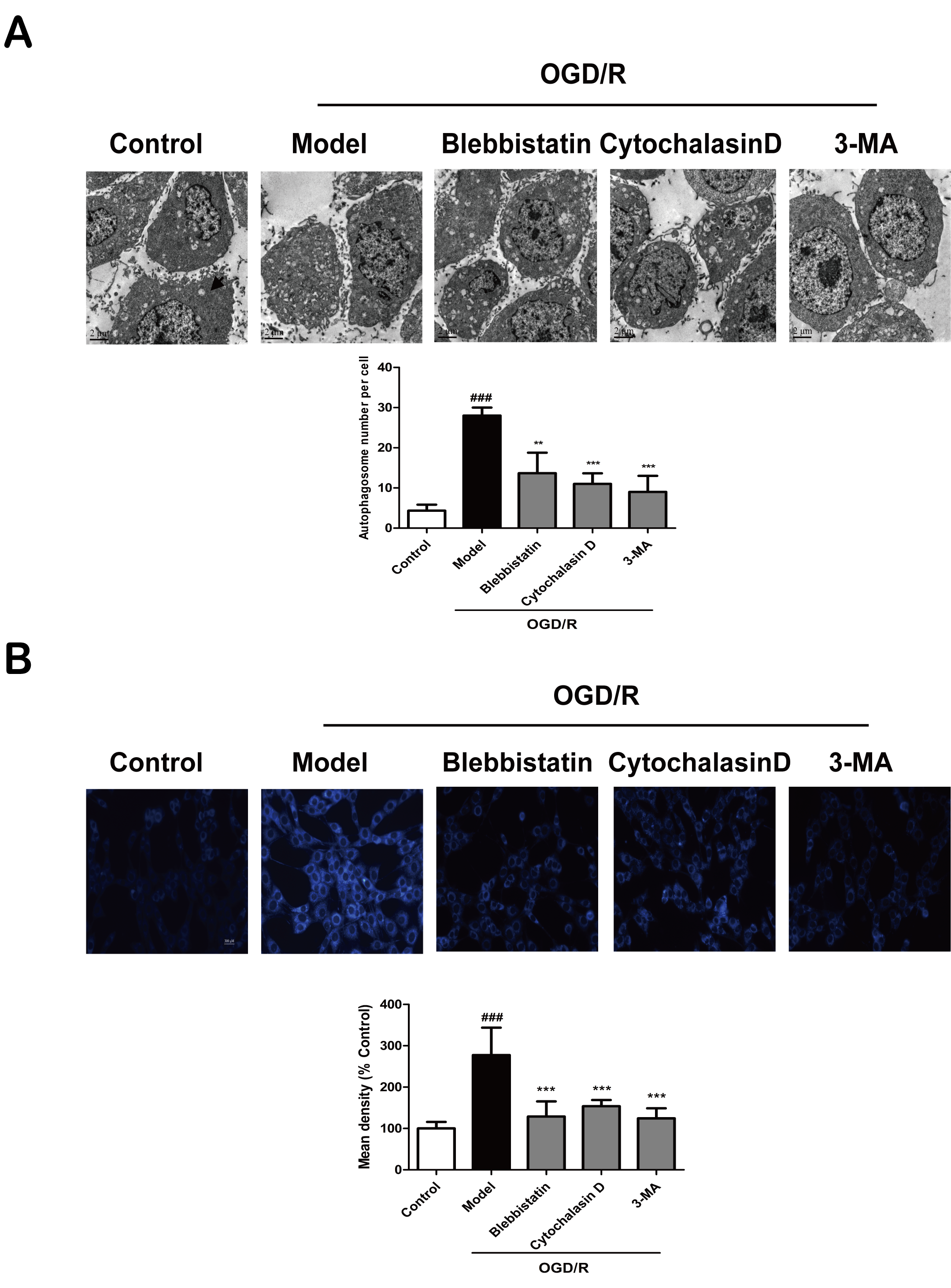

Supplement: Supplementary file 9 — Supplementary Figure 8 [file 41419_2020_2639_MOESM9_ESM.tif]

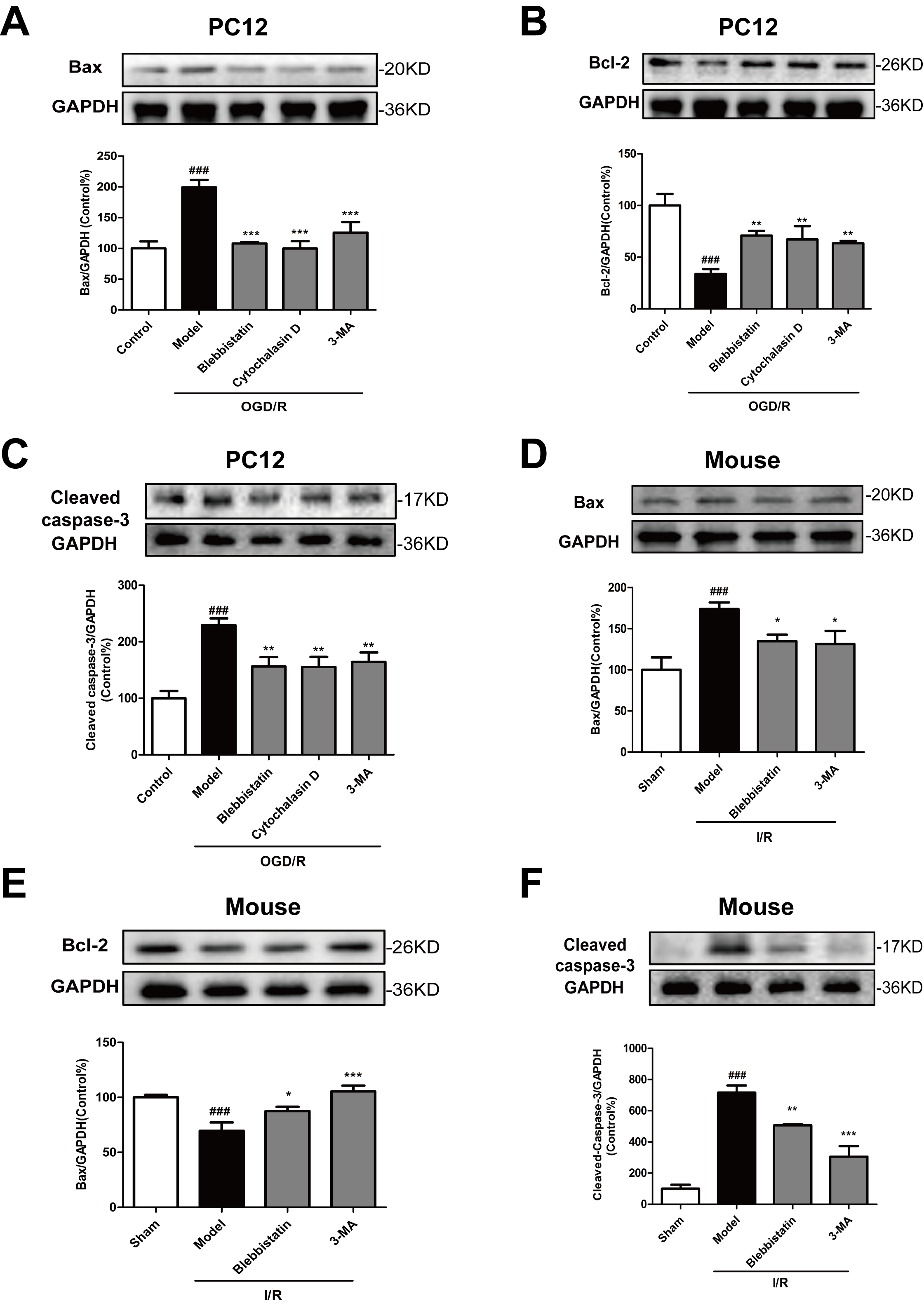

Supplement: Supplementary file 10 — Supplementary Figure 9 [file 41419_2020_2639_MOESM10_ESM.tif]

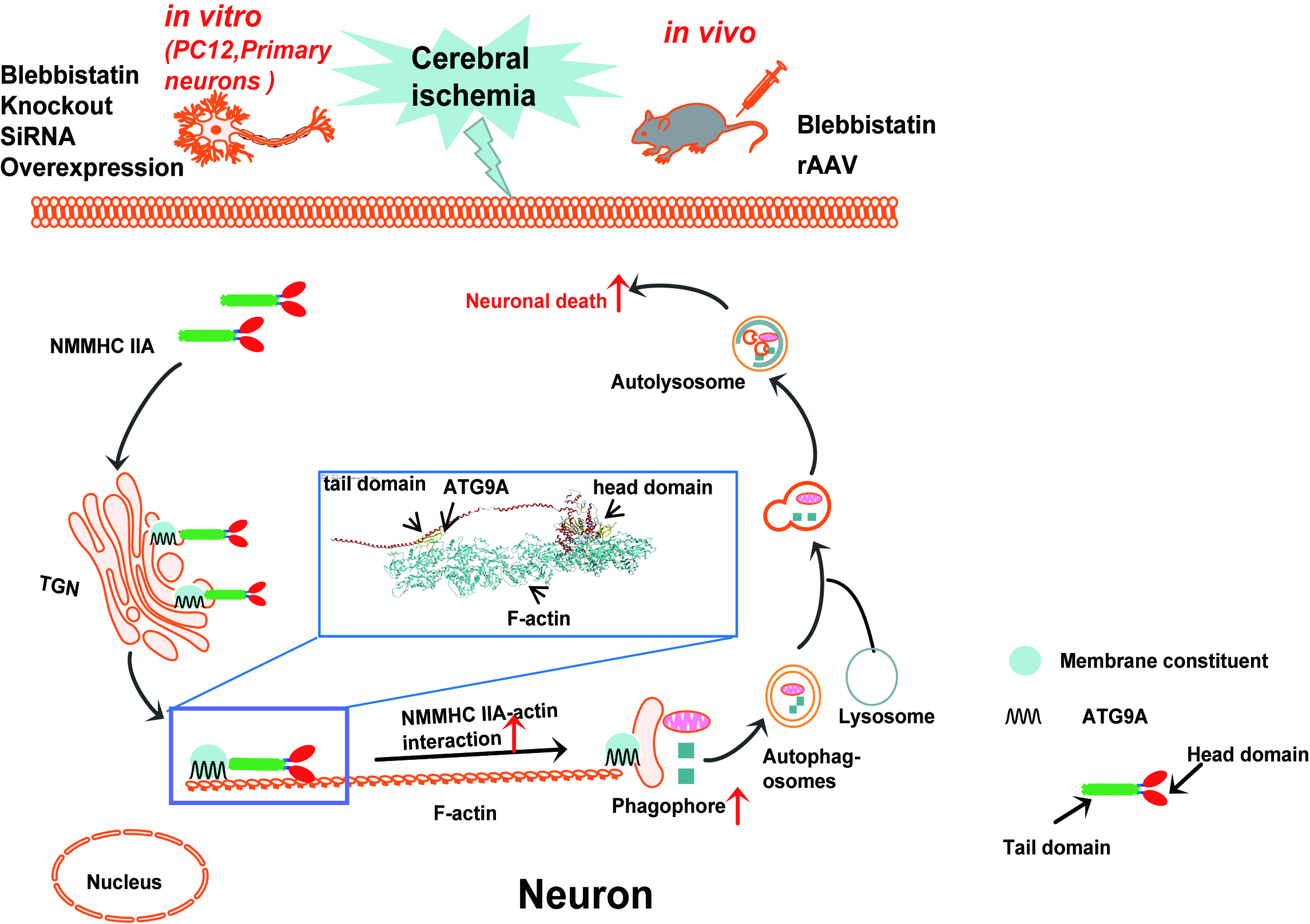

Supplement: Supplementary file 11 — Supplementary Figure 10 [file 41419_2020_2639_MOESM11_ESM.tif]
